# Systemic Nanomechanical Single‐Cell Profiling Reveals Mechanophenotype Transitions Under Therapeutic Perturbation

**DOI:** 10.1002/advs.76613

**Published:** 2026-07-17

**Authors:** Minhee Ku, Jinwon Kwon, Nara Yoon, Hyung Kwon Byeon, Jaemoon Yang

**Affiliations:** ^1^ Department of Radiology College of Medicine Yonsei University Seoul Republic of Korea; ^2^ Convergence Research Center for Systems Molecular Radiological Science Yonsei University Seoul Republic of Korea; ^3^ Department of Otorhinolaryngology‐Head and Neck Surgery Soonchunhyang University College of Medicine Seoul Republic of Korea

**Keywords:** cancer biomechanics, cytoskeletal nanostructure, mechanomics, mechanophenotype transition, multi‐nanoscopy, single‐cell nanometrology

## Abstract

Mechanical remodeling of cancer cells plays a critical role in regulating invasive behavior, yet its quantitative relationship with therapeutic response remains insufficiently defined. Here, we systematically characterize drug‐induced mechanophenotype changes at the single‐cell level using an integrated nanomechanical profiling approach that combines atomic force microscopy‐based force mapping of fixed cells, high‐resolution imaging, and cytomorphometric analysis under room‐temperature conditions. Pharmacological perturbation induces pronounced cytoskeletal reorganization accompanied by increased cortical stiffness and surface roughness. Systemic multivariate analysis identifies 11 biophysical parameters associated with invasive capacity, with nucleus modulus, cytoskeletal network density, and cortical roughness emerging as dominant contributors. Dimensionality reduction (principal component analysis (PCA) and partial least squares discriminant analysis (PLS‐DA)) reveals a distinct mechanophenotype transition characterized by elevated stiffness and suppressed protrusive activity. Especially, reduced invasiveness correlates with increased cortical roughness and reorganization of perinuclear cytoskeletal structures, indicating that these features define a quantitative mechanical signature of phenotypic reprogramming. This integrated mechanical signature enables discrimination between invasive and noninvasive states at the single‐cell level. These results establish nanomechanical profiling as a quantitative framework for assessing drug‐induced phenotypic transitions and provide a complementary approach to conventional molecular assays for evaluating therapeutic response in cancer cells.

## Graphical abstract

Single‐cell mechanomics demonstrates that pharmacological perturbation changes cytoskeletal and cortical structure, suppressing cancer cell invasiveness. By integrating atomic force microscopy (AFM)‐based cortical measurements with stimulated emission depletion (STED)‐resolved adhesion and cytoskeletal organization, this approach identifies nanoscale features, including surface stiffness and force transmission, as predictive biophysical markers of metastatic potential. In addition, we established a quantitative framework that links mechanophenotype with therapeutic response.

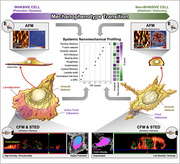

## 1 Introduction

Genetic profiling has long dominated cancer research. However, increasing attention is now being directed toward the interactions between cancer cells and the tumor microenvironment (TME), particularly regarding the physical properties of cells [1, 2]. Reflecting this shift in perspective, biophysical advances have enabled investigation of cancer cells and their dynamic interactions with stromal and immune components, which have become critical to understanding tumor progression. Despite these developments, the quantitative characterization of mechanical signaling between cells and the intrinsic physical properties of cancer cells remains limited [3]. While conventional genetic profiling has long guided our understanding of malignancy, it is increasingly evident that cytoskeletal organization and the resulting biophysical properties, such as cortical stiffness and surface roughness, modulate cell behavior in clinically meaningful ways. This recognition has prompted a reevaluation of standard cancer models and efficacy metrics, as morphological and mechanical reprogramming remain poorly quantified in drug response evaluations. Cytoskeletal networks undergo dynamic reorganization in response to genetic mutations or external stress, leading to changes in key biomechanical parameters, including cortical stiffness, surface roughness, and mechanical responsiveness. These properties are increasingly recognized as critical regulators of cellular invasiveness and metastatic potential [4, 5]. In previous work, we demonstrated that cell modulus‐based nanomechanical profiling enables quantitative assessment of cellular stiffness and its association with metastatic potential [6]. We observed that cancer cells exhibiting lower stiffness tended to be more invasive, and that these changes were closely associated with cytoskeletal remodeling, highlighting the potential of mechanical phenotyping as a functional readout of cancer cell state.

Nevertheless, conventional assessments of anticancer drug efficacy rely primarily on molecular or viability‐based endpoints, often overlooking drug‐induced cytoskeletal remodeling and the resulting mechanical transitions. Morphological changes in cancer cells arise from coordinated reorganization of cytoskeletal networks composed of microfilaments, intermediate filaments, and microtubules [7, 8]. These structural changes are not merely passive outcomes, but are intricately linked to signal transduction pathways, nuclear–cytoskeletal coupling, cell polarity, and focal adhesion dynamics [9]. Consequently, mechanical remodeling represents an integrated manifestation of intracellular signaling and structural adaptation, which remains insufficiently quantified in current therapeutic evaluation frameworks [10].

To overcome these limitations, we aimed to systematically investigate drug‐induced mechanophenotype transition using a cancer cell model characterized by therapeutic resistance and high invasiveness. BRAF^V600E^‐mutant cancer cells are a clinically important model in which targeted inhibition induces adaptive resistance and phenotypic plasticity [11, 12]. However, because these adaptive responses are often not captured by existing analytical methods, it is emphasized that an alternative quantitative framework is needed to analyze structural and mechanical remodeling at the nanoscale.

Recent shifts in mechanobiology have highlighted the mechanical interface between cells and their microenvironment in regulating cancer progression and therapeutic response. In this study, we thus establish a single‐cell nanomechanical profiling framework by integrating atomic force microscopy (AFM)‐based measurements of cortical mechanics with high‐resolution imaging of cytoskeletal organization. Especially, nanoscale biomechanical states can be directly quantified, and key physical parameters can be linked to cancer cell invasiveness and therapeutic response. Unlike conventional molecular analysis methods, this approach captures drug‐induced structural and mechanical remodeling that would otherwise remain unresolved, enabling a more quantitative interpretation of mechanophenotype transitions at single‐cell resolution.

## 2 Results and Discussion

### 2.1 Mechanical Polarization in Response to Therapeutic Perturbation

**FIGURE 1:**
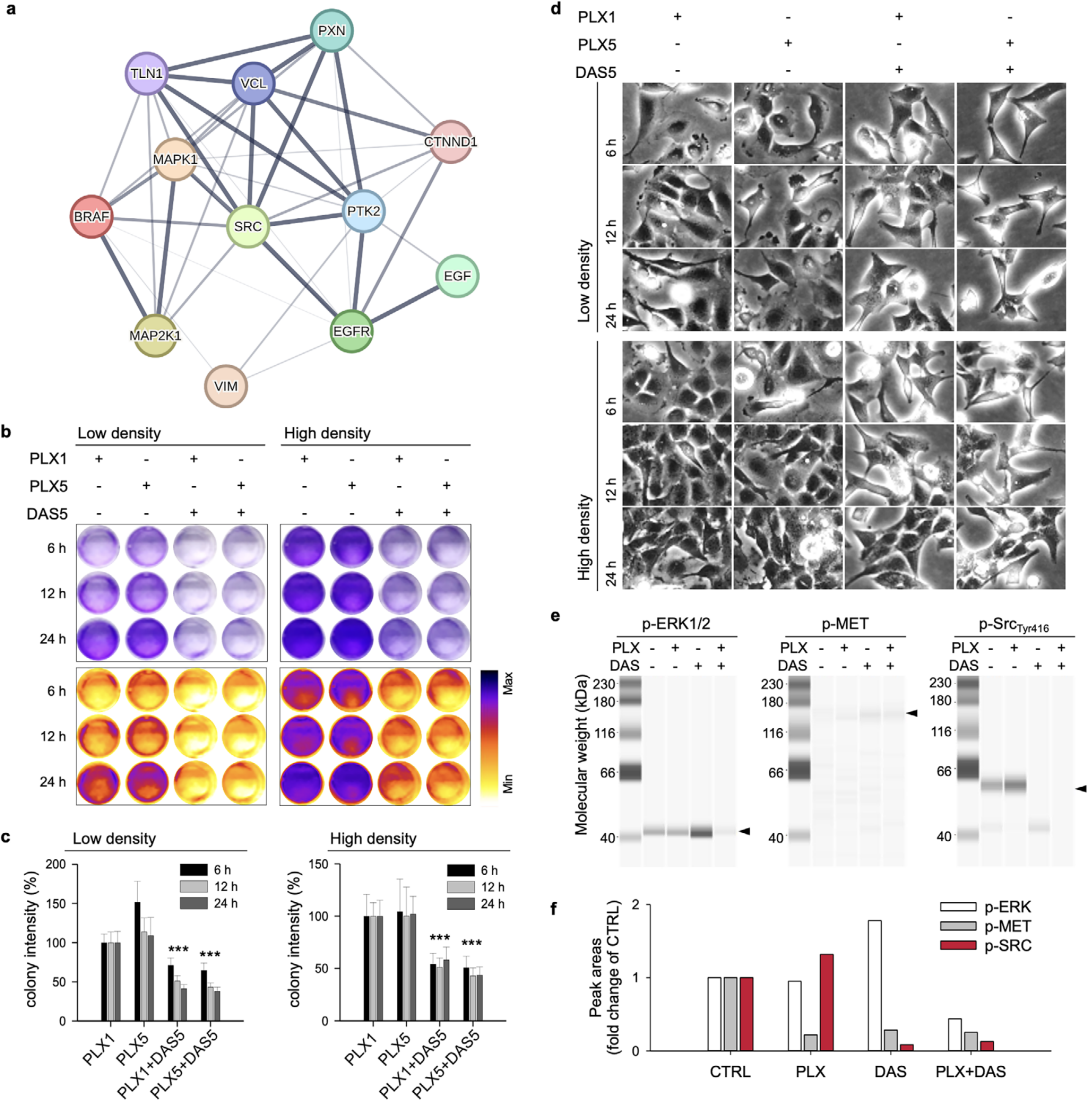
Therapeutic coinhibition suppresses proliferation and modulates EGFR signaling in BRAF^V600E^‐mutant cancer cells. (a) In silico protein–protein interaction network of cytoskeletal and focal adhesion‐related molecules functionally associated with BRAF signaling. (b) Exemplary crystal violet‐stained images demonstrating colony formation dynamics in 8505C anaplastic thyroid carcinoma cells treated with PLX4032 (abbreviated as PLX1 for 1 µm and PLX5 for 5 µm), dasatinib (DAS5; 5 µm), or their combination, under low (5 × 10^4^ cells per well) or high (1 × 10^5^ cells per well) seeding densities. Colony growth and cell attachment were assessed at 6, 12, and 24 h posttreatment. Pseudocolor enhancement using a yellow‐hot lookup table (LUT) was applied to visualize relative changes in colony intensity and spatial distribution. (c) Quantification of colony intensity from (b), normalized to the 6 h PLX‐only condition, at low (upper) and high (lower) seeding densities. Combination treatment resulted in a time‐dependent reduction of colony growth under both density conditions (mean ± S.D., *n* = 4). Statistical significance was determined using one‐way ANOVA followed by Tukey's multiple comparison test. ***p < 0.001. (d) Phase‐contrast microscopic images illustrating morphological alterations in response to drug treatment at 6, 12, and 24 h in cultures seeded at low and high densities. (e) Digital blot output of phosphorylated ERK1/2, MET, and SRC (Tyr416), detected using the Wes capillary‐based immunoassay system under identical treatment conditions. (f) Fold changes in phosphorylated ERK (p‐ERK), MET (p‐MET), and SRC (p‐SRC) levels indicate synergistic inhibition in response to therapeutic pressure. Peak area under the curve (AUC) was calculated for quantification.

To investigate adaptive responses to BRAF inhibition, we employed BRAF^V600E^‐mutant cancer cells as a model system, using 8505C cells as a representative cell line. BRAF is a serine/threonine kinase within the MAPK signaling pathway, and the BRAF^V600E^ mutation constitutively activates oncogenic signaling associated with cancer cell proliferation, survival, and adaptive resistance. PLX4032, also referred to as vemurafenib, is a selective inhibitor of mutant BRAF. PLX4032 treatment can induce resistance‐associated phenotypic and mechanical changes. First, the functional connectivity between BRAF signaling and the cytoskeletal regulatory network was analyzed. Network‐based bioinformatics analysis was performed using the STRING platform, a protein–protein interaction database to predict and visualize functional associations between proteins. Through this analysis, we identified functionally enriched interactions between the BRAF–SRC signaling axis and cytoskeletal effectors, including EGFR, vinculin, paxillin, and vimentin (Figure 1a). EGFR and SRC are key signaling regulators of cell survival, migration, and cytoskeletal remodeling. EGFR functions as a receptor tyrosine kinase, whereas SRC is a nonreceptor tyrosine kinase closely associated with focal adhesion signaling and actin cytoskeletal regulation. Functional enrichment analysis of Kyoto Encyclopedia of Genes and Genomes (KEGG) pathways and Gene Ontology (GO) terms, which annotate biological pathways and gene/protein functions, further substantiated these associations, revealing statistically significant clustering within cytoskeletal and adhesion‐related processes—including focal adhesion, adherens junctions, and actin cytoskeleton regulation (*p* < 0.05) (Table S1). This finding supports the critical role of structural and biomechanical alterations in determining drug responsiveness. Building on this framework, we established a model of combined inhibition targeting BRAF^V600E^ and SRC using PLX4032 (PLX) and dasatinib (DAS) to investigate the impact of drug resistance on the mechanical properties and viability of cancer cells.

To eliminate the confounding effects of cell‐to‐cell contact inhibition, we seeded cells at two different densities (5 × 10^4^ and 1 × 10^5^ cells) and assessed dose‐dependent cellular responses. For dose screening, cells were treated with PLX at 1 and 5 µm for 6, 12, and 24 h, and cell viability was measured using a crystal violet assay (Figure 1b). Unexpectedly, PLX monotherapy (5 µm) promoted cell viability rather than suppressing it, suggesting the emergence of paradoxical drug resistance mechanisms (Figure 1c). In contrast, combination treatment with PLX and DAS consistently suppressed cell growth regardless of cell density, PLX drug concentration, or treatment duration, relative to PLX monotherapy. Furthermore, PLX‐treated cells exhibited an increased fraction of dipolar morphology (Figure 1d). The acquisition of dipolar, elongated morphology is indicative of cytoskeletal polarization, frequently associated with enhanced protrusive activity and migratory potential, which are hallmarks of SRC‐mediated mechanotransduction [13, 14, 15, 16]. SRC is a key regulator of cancer cell growth and metastasis, and interacts with multiple cytoskeletal proteins, including focal adhesion kinase (FAK), vinculin, vimentin, and actin filaments [17, 18]. Considering the close relationship between cytoskeletal organization and cellular mechanics, recent studies have investigated its involvement in cancer cell progression and metastasis through changes in cell morphology and biomechanical properties [19, 20, 21].

Accordingly, to evaluate whether SRC activation acts as a resistance‐related mechanism in BRAF^V600E^‐mutant thyroid cancer, Western blotting was performed, confirming increased Tyr416 phosphorylation in PLX‐treated cells compared to control (Figure 1e,f and Figure S1). However, SRC activation was completely inhibited in both DAS and combination treatment. Paradoxically, DAS monotherapy led to hyperactivation of compensatory pathways involving MET and ERK1/2, implicating potential feedback signaling loops triggered by SRC inhibition [22, 23, 24, 25]. This observation supports the hypothesis that DAS monotherapy can trigger compensatory survival pathways, whereas co‐treatment with PLX effectively abolishes this feedback. These results suggest that combined inhibition using PLX and DAS represents a promising strategy to simultaneously suppress cellular survival and mechanical adaptation associated with drug resistance in BRAF^V600E^‐mutant 8505C cancer cells. These findings indicate that the observed mechanical adaptations arise from the integrated effects of growth inhibition, EGFR‐associated signaling, and morphodynamical changes.

### 2.2 Spatial Imaging of Cytoskeletal Remodeling Under Targeted Kinase Coinhibition

**FIGURE 2:**
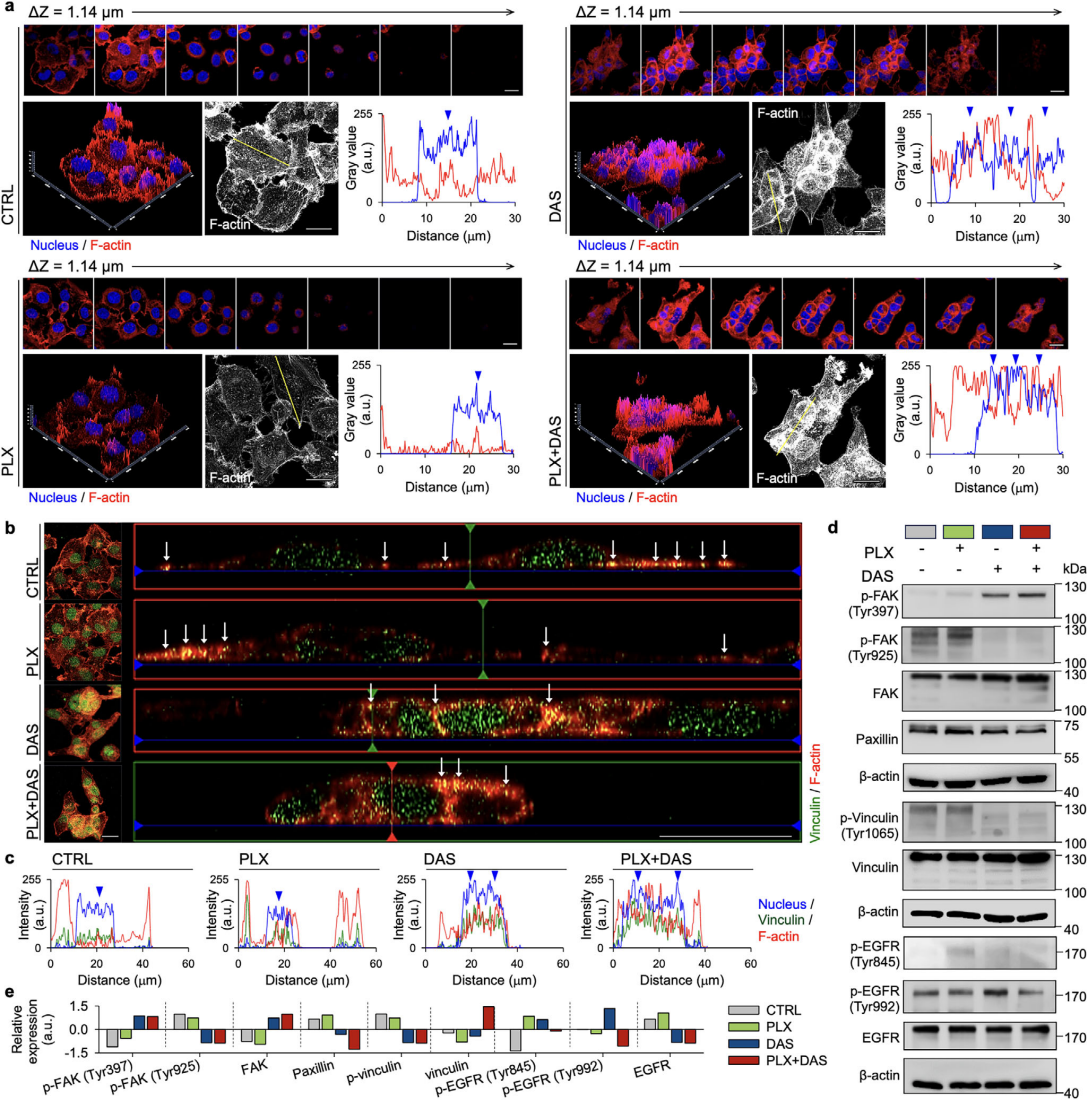
Therapeutic coinhibition of BRAF and SRC pathways disrupts actin cytoskeletal architecture and focal adhesion signaling in cancer cells. (a) Z‐stacked confocal microscopic images and the corresponding 2.5D surface renderings visualize the spatial distribution of F‐actin (red) and nuclei (blue) in 8505C cells following treatment. Line scan analysis across the indicated lines in grayscale projections quantifies the spatial separation between actin‐rich cortical regions and the nucleus. Blue arrowheads mark the nuclear position corresponding to fluorescence peaks. Each scale bar corresponds to 20 µm. (b) Orthogonal (*xz*‐plane) reconstructions depict the distribution of F‐actin (green), vinculin (red), and nuclei (blue) across treatment conditions. Colocalization of actin and vinculin at basal focal adhesions is highlighted in yellow (white arrows). Lateral projections were derived from *z*‐axis slices corresponding to the basal plane (blue line). Scale bars mean 20 µm. (c) Line scan intensity profiles along the apical–basal axis, extracted from orthogonal sections in (b), depict the relative distribution of actin (red), vinculin (green), and nuclei (blue). Blue arrowheads mark the nuclear peak. (d) Western blot analysis of the indicated focal adhesion‐related proteins. (e) Densitometric quantification of band intensities normalized to β‐actin, illustrating reduced activation of focal adhesion and EGFR signaling pathways under combined BRAF and SRC pathway inhibition.

To determine whether the morphological features of drug‐surviving cells reflect adaptive responses that underlie resistance, we analyzed the spatial distribution of actin, a central cytoskeletal component, following treatment. Leveraging z‐stacked confocal microscopy and 2.5D surface‐rendered maximum intensity projection (MIP) imaging, we visualized actin localization within the cell cortex and cytoplasm (Figure 2a). Along the *z*‐axis, PLX‐treated cells exhibited reduced vertical height, indicative of cell spreading, whereas DAS or combination treatment induced a more compact morphology with diminished lateral dimensions. In particular, actin accumulation at the cell cortex was enhanced in both the DAS and combination‐treated groups.

Quantitative line‐scan analysis across individual cells (Figure S2) confirmed distinct spatial patterns of actin distribution. In control cells, actin signals were diffuse and predominantly cytosolic, while in PLX‐treated cells, peripheral accumulation was noted despite an overall reduction in signal intensity, consistent with increased cell spreading. In contrast, DAS and combination treatment‐induced compact, clustered morphologies with strong perinuclear and pan‐cellular actin enrichment. This actin redistribution suggests treatment‐induced cytoskeletal remodeling that alters mechanical tension and cell–substrate interactions. Consistent with the established role of cytoskeletal reorganization in cellular stress adaptation, our data indicate that actin remodeling serves as a potential biomechanical correlation of drug response, positioning actin architecture as a mechanosensitive node mediating cell fate under targeted therapeutic stress [26, 27].

Cell migration relies on the dynamic formation of protrusions and the stabilization of substrate adhesion through focal adhesion complexes. At the leading edge, actin polymerizes while depolymerizing at the rear, defining directional movement [28, 29]. Vinculin orchestrates F‐actin organization within lamellipodia and mediates adhesion strength by bridging F‐actin and focal adhesion components [30]. Its activation also regulates retrograde F‐actin flow, which maintains structural integrity at the membrane interface [31]. To assess how drug treatments modulate cell migratory capacity, we analyzed vinculin–actin colocalization at focal adhesion sites using single‐cell confocal imaging (Figure 2b). In the lateral‐view images, where a blue line marks the bottom of the cell, drug‐induced morphological changes were distinguishable compared to the control. In nontreated control cells, colocalization signals (yellow spots) indicating vinculin–F‐actin colocalization were predominantly observed in the cell–substrate interface within the lamellipodium, whereas little signal was detected in the cytosol. In PLX‐treated cells, the lateral width was increased relative to the control, and the yellow dots at the lamellipodium appeared larger and brighter, suggesting enhanced focal adhesion complex formation. In contrast, DAS and combination treatment induced a marked morphological compaction and attenuated focal adhesion formation. F‐actin fibers exhibited poor alignment at the substrate interface, with reduced peripheral intensity and minimal lamellipodia formation, indicative of impaired cell–substrate adhesion and suppressed migratory potential. These findings suggest that SRC inhibition via DAS weakens cell–substrate adhesion while concurrently triggering compensatory reinforcement of intercellular junctions. The spatial redistribution of vinculin–F‐actin complexes toward cell–cell interfaces reflects a biomechanical transition that prioritizes collective cohesion over individual motility. Colocalization of vinculin and F‐actin at these junctions likely contributes to the stabilization of intercellular contacts and suppression of migratory behavior by maintaining junctional integrity [32, 33]. This enhanced cohesion within the cell cluster may, in turn, restrict autonomous cell movement. Furthermore, the near‐complete absence of colocalization signals at lamellipodia and substrate adhesion zones in combination‐treated cells underscores a broad disruption of both focal adhesion architecture and intercellular connectivity.

We subsequently examined the intracellular localization of vinculin and F‐actin using plot profile analyses, which revealed treatment‐dependent differences in focal adhesion organization (Figure 2c and Figure S3). In control and PLX‐treated cells, F‐actin localized predominantly at the distal ends of the cell body, whereas DAS‐ and combination‐treated cells exhibited perinuclear F‐actin accumulation. Especially, PLX‐treated cancer cells exhibited increased cell length, with regions of elevated F‐actin intensity closely aligned with areas of enhanced vinculin expression. These findings suggest that the paradoxically enhanced motility observed following PLX treatment is associated with strengthened focal adhesion formation, which is effectively suppressed under the combination treatment. While visualizing structural remodeling in terms of actin distribution and focal adhesion formation after drug treatment, we next investigated whether these morphological changes were mechanistically linked to the induction of cell death via suppression of drug resistance.

To elucidate the impact of drug treatments on focal adhesion signaling, we assessed the expression and phosphorylation states of key adhesion regulators and downstream EGFR effectors (Figure 2d and Figure S4) [34]. Phosphorylation at FAK Tyr397, a mechanosensitive autophosphorylation site linked to SRC activation and cell adhesion, was elevated following DAS or combination treatment [35, 36]. In contrast, phosphorylation of FAK at Tyr925, a paxillin‐binding site, promotes the formation of integrin‐induced FAK signaling complexes through the recruitment of Grb2 [37, 38, 39]. Tyr925 phosphorylation typically occurs in the later stages of FAK signaling and plays a key role in regulating cell motility by activating the Ras–MAPK pathway [40, 41]. After DAS or combination treatment, we observed an increase in Tyr397 phosphorylation alongside a marked reduction in Tyr925 phosphorylation, indicating a selective shift in FAK signaling dynamics. This pattern reflects activation of initial adhesion signals, while later‐stage pathways involved in cell motility are suppressed. Concurrently, paxillin expression was downregulated, consistent with reinforced substrate adhesion and enhanced cell–cell cohesion, which may underlie the reduced motility phenotype. Phosphorylation of vinculin at Tyr1065 by SRC kinase triggers a conformational shift that anchors F‐actin to the plasma membrane and reorganizes the actin cytoskeleton through engagement with the FAK complex [42, 43]. This posttranslational modification strengthens vinculin–actin binding, thereby stabilizing focal adhesions and facilitating directional cell migration. However, under DAS or combination treatment, Tyr1065 phosphorylation was markedly diminished, suggesting a breakdown in focal adhesion integrity and reduced mechanical signaling capacity. Phosphorylation of EGFR at Tyr845, a direct substrate of SRC kinase, initiates downstream SRC–STAT signaling that promotes cellular growth and metabolism [24, 44]. Tyr992 phosphorylation recruits PLCγ, activating the PLCγ–PKC axis involved in proliferation, motility, and differentiation [45]. Under combinatorial inhibition with PLX and DAS, phosphorylation at both sites was markedly reduced, indicating suppression of EGFR‐driven signaling cascades associated with survival and migration.

To quantify the treatment‐induced changes in mechanosignaling, we performed densitometric analysis of phosphorylation levels across key nodes, including EGFR, FAK, and vinculin (Figure 2e). These phospho‐sites function as convergence points linking growth factor signaling with adhesion‐dependent cytoskeletal remodeling. Their differential modulation across PLX, DAS, and combination treatment underscores their dynamic roles in coordinating cellular adaptations to external perturbations. Importantly, the phosphoprotein profiles reflected the morphological phenotypes observed by confocal microscopy, reinforcing the mechanistic association between actin remodeling, focal adhesion disruption, and drug‐induced suppression of motility and survival. This alignment supports the notion that coinhibition of BRAF and SRC pathways impairs critical prosurvival and promigratory signaling axes.

### 2.3 Therapeutic Disruption of Vimentin–Actin Polarity and Cell Migration Capacity

**FIGURE 3:**
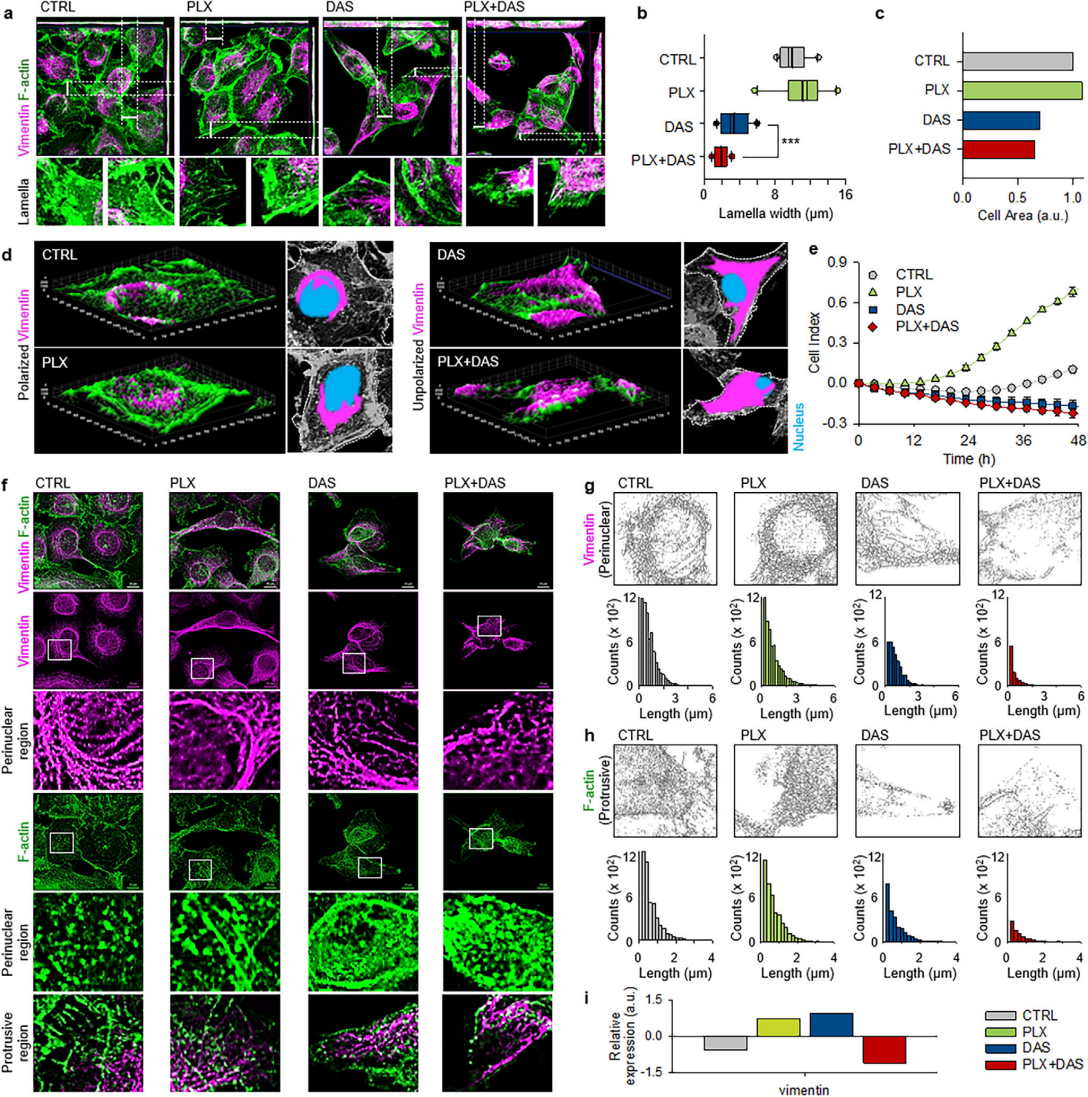
Therapeutic modulation of vimentin‐mediated cytoskeletal organization restricts lamellar protrusion and alters cell invasion. (a) Pseudocolor orthogonal reconstructions of vimentin (magenta) and F‐actin (green) illustrate lamellipodial architecture (white dashed lines). Insets emphasize peripheral actin distribution and lamella width. (b) Quantification of lamella width based on the distance from the perinuclear vimentin region to the leading edge. (c) Cell spreading area determined by projected actin footprint. Data are presented as mean ± SD, with *n* = 10 cells per condition. Statistical significance was determined using one‐way ANOVA followed by Tukey's multiple comparison test. ***p < 0.001. (d) 3D surface renderings (left) and the corresponding pseudocolored 2D projections (right) of vimentin (magenta) and nuclei (blue), highlighting perinuclear filament arrangement. (e) Real‐time cell analysis (RTCA) quantifying invasive behavior posttreatment. (f) High‐resolution STED images of vimentin (magenta) and F‐actin (green) in perinuclear and protrusive zones. Separate imaging regions highlight their distinct spatial patterns. Skeletonized images show cytoplasmic filament networks of (g) vimentin and (h) F‐actin, with histograms quantifying global filament length changes. (i) Densitometric quantification of vimentin expression levels normalized to β‐actin, based on Western blot analysis.

Cells integrate external mechanical forces through cytoskeletal networks to preserve structure and coordinate migration. Among these, intermediate filaments (IFs), particularly vimentin, form a resilient cytoplasmic scaffold linking the nucleus to the cell cortex, thereby maintaining cellular integrity, positioning, and polarity [46, 47, 48]. Given that lamellipodial dynamics and focal adhesion remodeling are hallmarks of migratory adaptation, it is plausible that IF networks undergo reorganization under therapeutic pressure. Vimentin, predominantly concentrated in the perinuclear zone, bridges actin and microtubule systems, orchestrating nuclear positioning and mechanical homeostasis [49]. Beyond structural roles, vimentin modulates actin‐based protrusions and contractile actomyosin networks at the leading edge [50]. Especially, its retrograde relocation during migration stabilizes actin arcs and reinforces front–rear polarity [51]. To investigate how therapeutic intervention alters this coordination, we visualized the spatial distribution of vimentin and F‐actin following treatment (Figure 3a). In control and PLX‐treated cells, vimentin localized perinuclearly, while F‐actin extended into lamellipodia. However, DAS and combination treatment induced ectopic vimentin dispersion toward the cortex and disrupted actin organization, suggesting polar destruction.

To assess treatment‐induced changes in leading‐edge architecture, we quantified lamella width at the single‐cell level, defined as the distance from the perinuclear vimentin region to the cell periphery (white lines in Figure 3a). As shown in Figure 3b, PLX‐treated cells exhibited significantly broader lamellae compared to control, consistent with enhanced protrusive activity. By contrast, DAS or combination treatment led to irregular vimentin bundling extending into the leading edge, effectively compressing the lamellipodial space. These structural changes were statistically significant, with both DAS‐containing groups showing a marked reduction in lamella width. High‐magnification imaging further confirmed a loss of organized F‐actin architecture and a concomitant increase in cortical vimentin signal intensity in these treatment conditions (Figure 3a). Consistent with the reduction in focal adhesion observed in Figure 2, the projected cell area was expanded following PLX treatment but significantly diminished under DAS or combination treatment (Figure 3c).

Drug‐induced reorganization of the vimentin IF network substantially influences lamellipodia formation and cellular polarity. Previous studies have demonstrated that pharmacological perturbation of vimentin architecture promotes perinuclear bundling, thereby regulating actin filament flow and stabilizing nuclear position [52]. Conversely, when vimentin filaments become dispersed throughout the cytoplasm rather than confined to the perinuclear zone, their interactions with actin become disrupted, potentially compromising cytoskeletal integrity and impairing directional migration. As shown in Figure 3d, control and PLX‐treated cells maintained strong vimentin enrichment near the nucleus, reflecting a preserved polarized architecture. In comparison, DAS or combination treatment led to reduced perinuclear concentration and lateral spreading toward the cell periphery of vimentin. This spatial redistribution disrupts the structural balance of actin filaments, interfering with lamellipodia formation and focal adhesion remodeling, ultimately reducing cell motility and invasiveness. Thus, both DAS‐ and combination‐treated groups displayed significantly reduced motility and invasive capacity.

To evaluate whether the observed reduction in motility is mechanistically associated with alterations in the vimentin–actin network and defective lamellipodia formation, which are both essential for protrusion and invasion, we performed a transwell‐based invasion assay (Figure 3e). Cells were seeded in the upper chamber with serum‐free medium, while the bottom chamber contained 10% FBS as a chemoattractant. Cellular invasiveness was quantified by monitoring real‐time cell index values, which reflect impedance changes as cells penetrate through the gel layer and adhere to the bottom membrane surface. PLX‐treated cells exhibited significantly enhanced invasive migration relative to the control group, likely due to increased lamellipodia formation and strengthened focal adhesion assembly. By comparison, DAS‐ and combination‐treated cells exhibited minimal invasion, suggesting that disruption of the vimentin–actin network is tightly linked to the suppression of invasive potential. Redistribution of vimentin from the perinuclear zone to the broader cytoplasm constrains the spatial domain necessary for lamellipodia expansion. The resulting structural reorganization is likely to perturb actin filament dynamics, thereby compromising both cell migration and invasive behavior.

To more precisely visualize cytoskeletal remodeling, we employed stimulated emission depletion (STED) microscopy for high‐resolution imaging. This technique surpasses conventional confocal microscopy in resolution, enabling visualization of cytoskeletal ultrastructure with sub‐30 nm precision [53, 54]. Such resolution is critical for characterizing the intricate architecture and dynamic remodeling of cytoskeletal elements [55, 56]. STED imaging enabled detailed analysis of the perinuclear distribution of vimentin and the organization of actin filaments at protrusive sites (Figure 3f). In cells treated with PLX, vimentin filaments exhibited compact, coherent organization around the nucleus. Conversely, combination‐treated cells exhibited dispersed vimentin networks or punctate aggregates, suggesting compromised cytoskeletal stability. The perinuclear actin cap, a specialized assembly of actin filaments spanning the nuclear region, has been shown to regulate nuclear positioning and mechanotransduction [57]. It also contributes to establishing cellular polarity and coordinates with stress fibers and focal adhesions to regulate nuclear translocation during migration [58, 59]. In PLX‐treated cells, actin filaments formed thick perinuclear bundles, and vimentin retained a well‐organized perinuclear distribution. This organization is indicative of a preserved perinuclear actin cap, supporting both nuclear positioning and directed migration. These findings suggest that alignment of the actin cytoskeleton with focal adhesions in PLX‐treated cells facilitates enhanced migratory behavior observed in functional assays.

In DAS‐ and combination‐treated cells, the perinuclear actin cap appeared structurally disrupted. As shown in the side‐view images (Figure 2b), actin filaments were disorganized and formed punctate aggregates above the nucleus, rather than surrounding it. This aberrant organization suggests a loss of connectivity between focal adhesion‐linked actin networks and the perinuclear region. This decoupling likely reduces filament tension and actomyosin contractility, thereby impairing perinuclear cap formation and directional migration. Vimentin filaments were similarly disrupted, exhibiting diffuse cytoplasmic dispersion rather than perinuclear enrichment. While PLX‐treated cells retained structural integrity conducive to migration, DAS or combination treatment resulted in cytoskeletal destabilization and diminished migratory capacity. These findings were further corroborated by high‐resolution STED imaging. In control and PLX‐treated cells, actin‐rich lamellipodia were preserved, and vimentin maintained spatial alignment with actin filaments, reflecting intact cytoskeletal architecture. These observations indicate that PLX preserves coordinated vimentin–actin interactions and focal adhesion integrity, thereby sustaining directional motility. In contrast, DAS and combination treatment led to a loss of actin‐based protrusions, accompanied by reduced peripheral F‐actin intensity. Vimentin filaments extended aberrantly toward the cell edge, independent of protrusive structures, indicative of impaired cytoskeletal coordination.

Quantitative analysis revealed significantly shortened branched filament lengths for both vimentin and F‐actin in DAS‐ and combination‐treated cells (Figure 3g,h). This global cytoskeletal disorganization compromises the structural framework required for cell motility, leading to impaired protrusion formation and reduced migration. Confocal microscopy lacks the resolution necessary for accurately discerning subcellular protein localization. Localized signal amplification may be misinterpreted as increased protein abundance, rather than reflecting regional filament bundling. In DAS‐ and combination‐treated cells, peripheral vimentin intensity appeared elevated (Figure 3a,d), potentially confounding interpretation as increased expression. High‐resolution STED imaging revealed that the apparent signal increase stemmed from abnormal cytoplasmic redistribution of vimentin, not from elevated protein levels (Figure 3f). Supporting this, Western blot analysis showed a significant decrease in total vimentin expression in the combination‐treated group (Figure S4). This reduction was corroborated by standardized densitometric quantification (Figure 3i). Taken together, the differential intracellular distribution and spatial organization of vimentin and actin filaments underlie the observed variations in cell migratory capacity. These findings suggest that cancer cells perceive and transduce mechanical cues at the cortex through coordinated vimentin–actin crosstalk. This interplay facilitates dynamic remodeling of the cytoskeletal scaffold and nuclear positioning, thereby governing directional migration. Collectively, our data position the vimentin–actin interaction as a critical determinant of both cellular mechanotype and invasive behavior, highlighting its potential utility as a mechanobiological indicator of metastatic competence.

### 2.4 Cortical Surface Instability as a Mechanical Readout of Therapeutic Response

**FIGURE 4:**
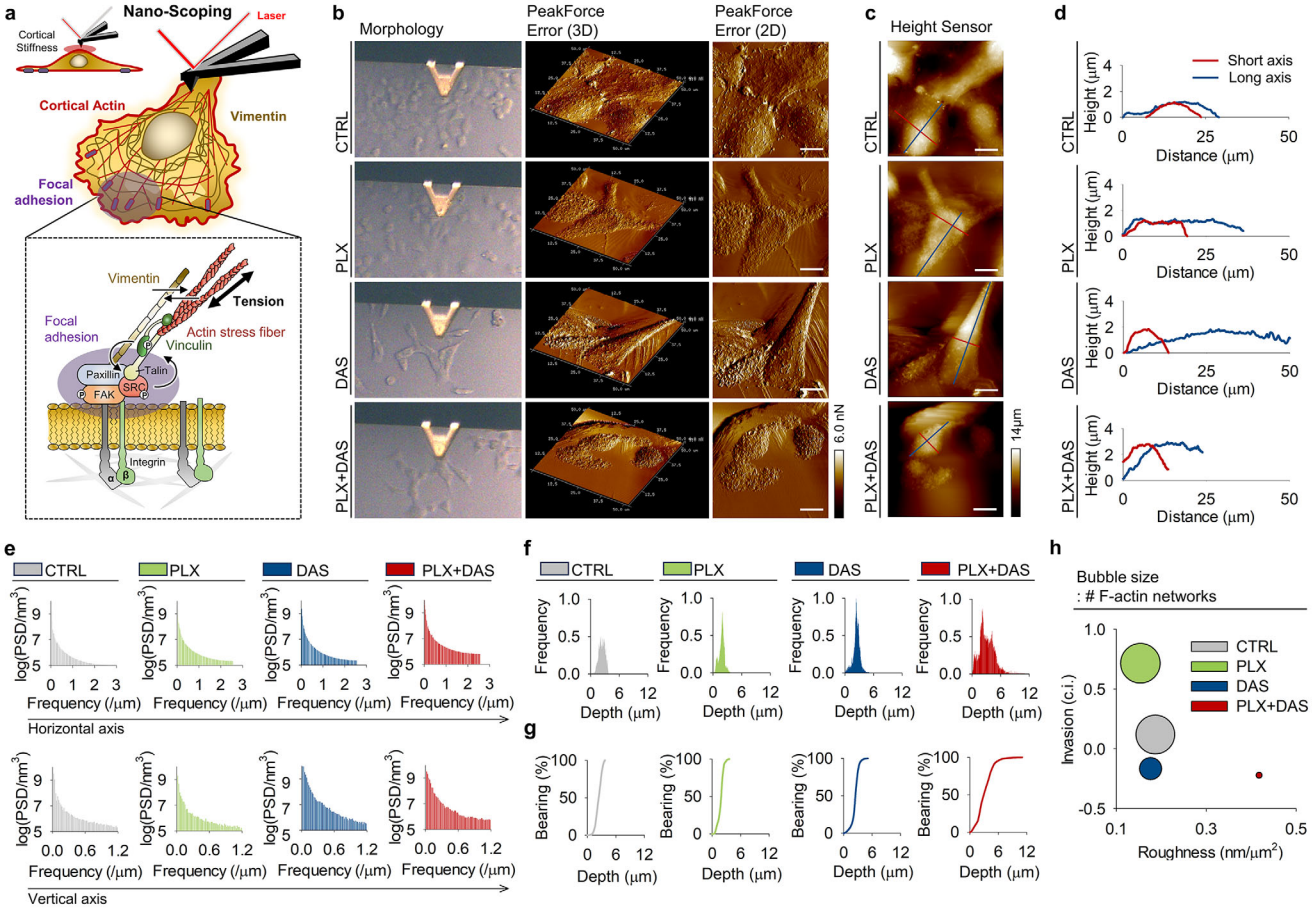
AFM‐based nanomechanical profiling reveals therapy‐induced alterations in surface stiffness and topographical complexity. (a) Schematic depicting aqueous‐phase Nano‐Tipping for nanomechanical profiling via AFM‐based Sneddon modulus analysis. (b) Phase‐contrast microscopic image indicating the AFM tip position on 8505C cells, accompanied by representative 3D‐rendered and 2D PeakForce Error maps acquired after treatment with PLX, DAS, or their combination. (c) Height sensor images and (d) the corresponding cross‐sectional profiles along short (red) and long (blue) axes illustrate topographical variation of the cellular surface. (e) Power spectral density (PSD) plots characterizing spatial roughness distributions along horizontal (upper) and vertical (lower) axes. (f) Histograms of surface indentation depths and (g) Bearing area curves computed from topographic depth profiles quantify surface irregularity. (h) Bubble plot integrating roughness, F‐actin network density, and invasive potential across drug treatment groups, highlighting mechanical and structural phenotypes associated with therapeutic response.

We subsequently investigated whether drug‐induced cytoskeletal remodeling translates into quantifiable alterations in the mechanical properties of individual cells. To address this, we employed a Nano‐Tipping strategy for single‐cell mechanical profiling, which permits the assessment of cell stiffness under aqueous conditions and thereby provides a physiologically relevant measure of drug‐induced biomechanical changes (Figure 4a). AFM enables precise analysis of nanomechanical parameters such as height, modulus, adhesion, dissipation, and deformation. In particular, the PeakForce Quantitative Nanomechanical Mapping (QNM) mode operates in a gentle tapping regime that minimizes lateral force and mechanical perturbation to biological samples, making it suitable for high‐resolution nanomechanical mapping of cellular surfaces [60, 61]. Compared with conventional AFM imaging, PeakForce QNM offers a faster acquisition rate and reduced artifacts from sample deformation, thereby enabling more accurate submolecular quantification of mechanical properties [62]. Surface stiffness represents a critical mechanical property that directly influences cell motility and invasiveness through its linkage to cytoskeletal organization. During cancer cell migration and invasion, physical remodeling of the cell surface occurs and profoundly affects both morphology and mechanical behavior [63, 64, 65]. Quantitative assessment of surface stiffness following drug treatment, therefore, provides a robust approach to evaluate the therapeutic modulation of metastatic potential. Our previous work established a strong correlation between cell surface stiffness and metastatic competence in cancer cells [6].

Here, we specifically investigated how the tested drug regimens influence surface mechanical properties to evaluate the efficacy of the combination therapy in suppressing prometastatic traits. Surface stiffness measurements were performed using PeakForce QNM scanning with a sharp cantilever tip of 0.1 N m^−1^ spring constant. In cell mechanics research, cantilever stiffness typically ranges from 0.01 to 0.6 N m^−1^, and the use of sharp tips enables simultaneous mapping of localized nanomechanical properties and high‐resolution topography [66, 67]. This setup allowed us to perform both 2D and 3D PeakForce Error imaging to precisely characterize drug‐induced morphological changes and quantify nanoscale alterations in surface structure. A representative screenshot showing the AFM cantilever positioned above individual cells, together with the corresponding PeakForce Error images, provided enhanced visualization of cellular morphological features (Figure 4b). The peak force error signal, which is derived from the combined analysis of deflection errors in contact mode and amplitude error in tapping mode, is particularly effective for reconstructing the 3D topography of the cell surface. In untreated control cells, rounded nuclei and cytoplasmic regions closely adherent to the substrate were clearly distinguishable, with cell–cell borders remaining largely intact. By comparison, PLX‐treated cells exhibited a more stretched morphology accompanied by a noticeable reduction in cell–cell contact areas. DAS‐treated cells displayed pronounced elongation and stretching, with localized increases in surface height within extended regions. Especially, cells subjected to the combination treatment presented globally contracted shapes and weakened substrate adhesion, occasionally complicating image acquisition. These observations indicate that modulation of SRC signaling exerts a critical influence on cytoskeletal organization and associated structural remodeling.

Remodeling of the cortical actin network is likely accompanied by global changes in cell morphology and surface complexity. To quantitatively assess these alterations, we performed line profiling along the red and blue transects marked on individual cells in the AFM height images (Figure 4c). As shown in Figure 4d, control cells exhibited a cobblestone‐like appearance with profile line lengths ranging from 20 to 30 µm. Height profiles consistently showed elevated regions corresponding to the nucleus. In contrast, cells treated with PLX or DAS displayed blurred boundaries between the nucleus and cytoplasm and adopted elongated, spindle‐like morphologies. The relative distance of the two analyzed lines increased 1.5‐ to 2‐fold in the PLX‐treated group and over threefold in the DAS group. Cells treated with combination therapy exhibited the most compact and contracted morphology among all groups. In addition, waveform analysis of the profile curves revealed that drug‐treated cells exhibited shorter wavelengths and increased amplitudes compared to controls, indicating concurrent changes in cell shape and surface microtopography. To further quantify these structural differences, we performed power spectral density (PSD) analysis, depth histogram profiling, and bearing area calculations, which together characterize height distribution and surface complexity. PSD analysis, which evaluates energy distribution in the frequency domain, provides insights into surface roughness and structural periodicity. The frequency domain consisted of two dominant waveforms, horizontal (long‐period) and vertical (short‐period) components (Figure 4e). PLX‐ and DAS‐treated cells exhibited slightly reduced PSD values compared to control, indicating diminished specific structural periodicity at the surface. However, the combination‐treated cells exhibited the highest overall PSD values among all conditions. Remarkable changes in the low‐frequency region suggest that the long‐wavelength periodic surface patterns are strengthened. Such structural changes were expected to influence surface height distributions as well. Therefore, we performed histogram analyses of depth distributions across the entire cell surface (Figure 4f). Control cells displayed a relatively uniform depth distribution within the 1–3 µm range, reflecting minimal surface structural changes. In PLX‐treated cells, the histogram shifted leftward, indicating surface flattening with reduced roughness and structural rigidity. This observation aligns with the loss of specific surface patterns identified in the PSD analysis and reflects simplification of fine surface architecture. DAS‐treated cells exhibited an expanded depth distribution ranging from 1 to 6 µm, suggesting the presence of deeper surface indentations in localized areas. Combination‐treated cells exhibited the widest depth range (2–12 µm), characterized by heterogeneous and irregular surface features that were not observed under other conditions.

Subsequently, we quantitatively evaluated how changes in surface depth influence overall cell morphology through surface profile analysis. Bearing area analysis represents area ratios at specific depths (Figure 4g). Comparing the bearing curve shapes and the depths reached at 100% provided insights into surface roughness and structural complexity across treatment conditions. Typically, a steeper curve reflects a relatively flat surface with minimal depth variation, while a more gradual slope indicates the presence of diverse depth structures. In control, the curve reaches 100% at shallow depths, suggesting a relatively smooth surface with little structural variation. Both PLX and DAS‐treated cells displayed similar patterns to the control, though with some localized depth variations. The combination‐treated cells reached 100% at approximately 12 µm in depth and indicated the presence of larger and more diverse surface features compared to other conditions. These results suggest that the combination treatment contributed to increasing surface complexity and the depth variation of surface structure. Overall, PLX treatment reduced surface roughness but slightly increased microirregularities relative to the control. DAS treatment led to surface irregularity and the formation of deeper structures in certain areas. Combination treatment resulted in the most pronounced increase in surface complexity, characterized by the development of diverse depth patterns. Collectively, PLX and DAS combination treatment altered the morphology and surface patterns of cells, leading to increased structural complexity and roughness frequency. These results underscore the ability of drug treatment to remodel cellular structure, extending beyond morphological changes to encompass alterations in surface complexity and heterogeneity. To further investigate the relationship between these structural changes and cancer cell behavior, we analyzed the correlations among invasive potential, protrusive F‐actin network, and surface roughness using a bubble plot (Figure 4h). PLX‐treated cells were positioned in the region characterized by high protrusive F‐actin network complexity and low surface roughness, consistent with their increased migratory and invasive capacity. In contrast, DAS‐ and combination‐treated cells exhibited reduced protrusive F‐actin network and lower surface roughness, coinciding with markedly suppressed invasiveness. These findings suggest that cytoskeletal reorganization and surface physical properties are closely linked to regulating cancer cell motility and invasion. In particular, in the combination‐treated group, the collapse of F‐actin networks and decreased surface roughness likely contributed to the pronounced inhibition of cell invasion.

### 2.5 Single‐Cell Nanomechanical Profiling Reveals Microvilli‐Driven Cortical Stiffening Upon Combination Therapy

Beyond global alterations in surface morphology, we further analyzed nanoscale features to elucidate the structural determinants underlying surface roughness. These variations in roughness are largely governed by the organization and morphology of fine cortical structures, particularly microvilli. Microvilli are finger‐like membrane protrusions composed of bundles of approximately 40 uniformly polarized actin filamentsthat increase the effective surface area and facilitate cellular absorption and secretion [68]. In malignant cells, structural abnormalities in microvilli, such as irregular morphology and elevated density, are closely associated with increased metastatic capacity [69]. Given that mechanical stiffness is closely linked to cortical cytoskeletal integrity, microvillar architecture may serve as a critical indicator of metastatic capacity and cellular aggressiveness.

**FIGURE 5:**
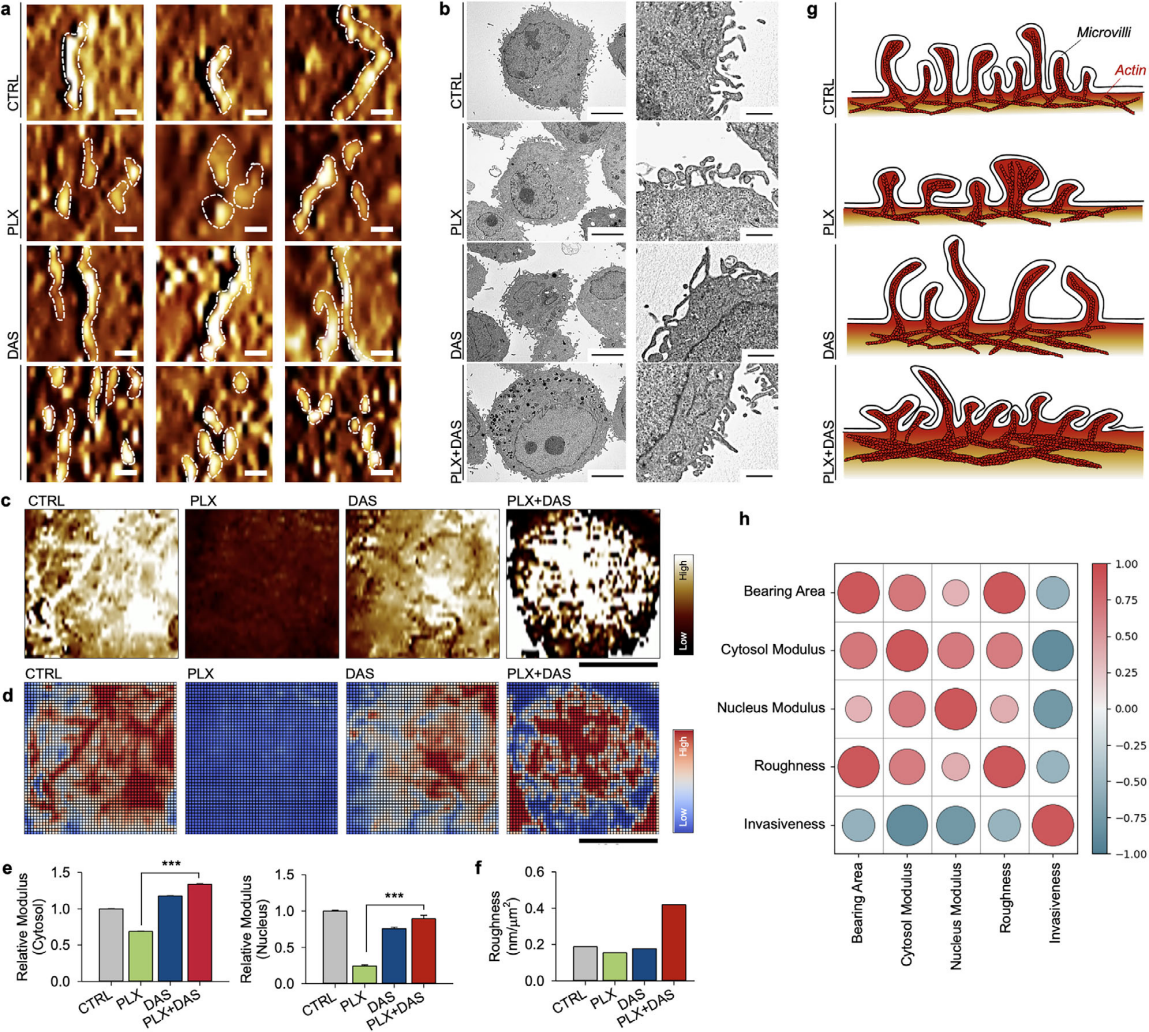
Therapeutic pressure modulates apparent cellular stiffness and surface roughness. (a) High‐resolution AFM topography of the apical membrane surface, derived from PeakForce error signal acquisition, delineating the nanoscale architecture and spatial distribution of microvilli. Scale bars mean 780 nm. (b) EM images verifying the presence of apical microvilli and surface complexity. Scale bars mean 5 µm. Enlarged right panels present membrane‐rich protrusive structures. Scale bars mean 1 µm. (c) Representative PeakForce QNM‐derived modulus maps of fixed cells under each treatment condition. The modulus values were interpreted as model‐dependent apparent Sneddon modulus values obtained under standardized AFM acquisition and fitting conditions. Scale bar indicates 10 µm. (d) Spatial map of the local Sneddon elastic modulus distribution, showing the treatment‐dependent differences in stiffness heterogeneity within the cell region. (e) Bar graphs compare cytosolic (left) and nuclear (right) apparent Sneddon modulus values based on cell‐restricted ROIs, indicating relative changes in cellular mechanical response among treatment groups. Data are presented as mean ± SD, with *n* = 10 cells per condition. Statistical significance was determined using one‐way ANOVA followed by Tukey's multiple comparison test. ***p < 0.001. (f) Quantitative assessment of cortical roughness based on AFM topographic profiles. Roughness was defined as the arithmetic mean of absolute height deviations. (g) Schematic illustrations summarize microvillar architecture, actin filament arrangement, and protrusion density across treatment groups. (h) Circular heatmap presenting correlations between nanomechanical features (Bearing Area, Cytosol Modulus, Nucleus Modulus, Roughness) and cell invasiveness.

To investigate whether drug‐induced changes in surface roughness were related to the reconstruction of microvilli structure, we first analyzed the protrusive surface structures using AFM Peak Force Error imaging (Figure 5a). AFM images were obtained by cropping and magnifying the Peak Force Error images, with representative protrusions indicated by white dashed outlines. In control cells, these protrusive structures exhibited varying lengths exceeding 2–3 µm and maintained a thick, linear morphology with well‐defined organization. In contrast, PLX‐treated cells showed a marked decrease in the height difference of protruding structures relative to the surrounding cell surface, as well as a decrease in overall structural complexity. DAS‐treated cells retained protrusive structures with a thickness comparable to that observed in control cells, but these structures tended to be longer and less densely distributed. In the combination treatment cells, protrusive structures appeared shorter, thicker, and more irregular, with a complex distribution pattern.

To determine whether these AFM‐detected protrusive structures corresponded to microvillar membrane structures, transmission electron microscopy (TEM) was performed to examine cell‐surface ultrastructure under each condition (Figure 5b). TEM is widely used to identify the ultrastructure of microvilli based on their characteristic finger‐like membrane morphology, and previous AFM‐based studies have also reported spatial correspondences between protrusive structures detected by AFM and Ezrin or Villin‐positive microvilli [70, 71]. In this study, TEM images clearly revealed finger‐like membrane protrusions extending from the cell surface, supporting the assignment of the AFM‐detected protrusive topography as microvillar membrane structures. Control cells displayed diverse microvillar structures, whereas PLX‐treated cells exhibited shorter, thicker, and less prominent microvillar structures. DAS‐treated cells showed fewer but elongated microvilli, while the combination‐treated cells were characterized by predominantly short and irregularly shaped microvilli. These complementary AFM and TEM analyses demonstrate that drug treatment induces reproducible remodeling of microvilli structures, which is associated with the observed changes in nanoscale surface roughness. Additionally, intracellular autophagosome formation was examined as a potential indicator of drug effects. While no significant differences were observed between control and PLX‐treated cells, autophagosome accumulation was markedly elevated in the combination‐treated group (Figure S5), suggesting enhanced cellular stress and potential activation of autophagic pathways.

Drug‐induced remodeling of microvilli morphology is likely to influence the mechanical properties of the cell cortex. This relationship was evaluated using Single‐Cell Nanoscoping to measure the apparent Sneddon modulus (Figure 5c). PeakForce Quantitative Nanomechanical Mapping (PF‐QNM) was used as a high‐resolution AFM‐based approach for spatially mapping local mechanical responses on surfaces by controlling nanometer‐scale probe indentation. Since the measurements were performed on chemically fixed cells and analyzed using the Sneddon contact model, the results represent the apparent Sneddon elastic modulus values that depend on the model under current PF‐QNM conditions, rather than the intrinsic physiological Young's modulus values of living cells [72]. The measured apparent modulus is expected to comprehensively reflect the mechanical response of the cell membrane, membrane‐associated cytoskeletal cortex, and underlying intracellular structures within the indentation volume. This interpretation is consistent with the reported cortex thickness of approximately 150–300 nm [73]. The cell cortex, composed of a contractile actin meshwork, plays a critical role in maintaining cell shape and regulating movement, division, and overall cellular mechanics. Because the cell cortex lies near the resolution limit of conventional light microscopy, high‐resolution nanomechanical techniques such as AFM provide complementary information for structural and mechanical characterization [74, 75].

We visualized a mechanical contrast between the glass substrate and the cell‐related region through PF‐QNM‐based Sneddon elastic modulus imaging. The brightest areas in the images correspond to the glass substrate, which exhibited markedly higher apparent stiffness (Figure S6). In contrast, the cellular regions represented lower apparent Sneddon modulus values than the surrounding substrate. For comparison between treatment groups, the same indicator scale was applied, and the relatively brighter intracellular regions were interpreted as regions with higher apparent stiffness under the same acquisition and analysis conditions. In the cell‐related regions, the nucleus and perinuclear areas generally showed higher apparent modulus values than the cytoplasmic area. This regional difference may reflect the contribution of nuclear‐associated mechanical support, including the nuclear envelope and nuclear lamina, and perinuclear cytoskeletal organization [76, 77].

The modulus heatmap of the nucleus and perinuclear region (Figure 5d) and the comparison of the corresponding regions (Figure 5e) revealed treatment‐dependent differences in apparent Sneddon modulus distribution. Control cells showed a relatively higher apparent modulus in the nucleus and perinuclear region than in the cytosol region. PLX‐treated cells exhibited significantly reduced apparent modulus across cellular regions, suggesting that the cell‐related mechanical state became softer. These observations are consistent with the increased invasiveness shown in Figure 3e and with previous studies reporting that nuclear softening in malignant cells enhanced deformability during invasion. In contrast, DAS‐treated cells exhibited higher apparent modulus than PLX‐treated cells, and the regions of high modulus were most prominent across the entire cell region in the combination of PLX and DAS. Taken together, these results suggest that PLX induces a relatively softened apparent mechanophenotype, whereas DAS‐containing treatment conditions appear to counteract this shift and promote a stiffer cell‐associated mechanical response. The observed stiffness redistribution was further supported by AFM height‐based surface roughness analysis (Figure 5f), highlighting the relationship between microvilli‐associated surface remodeling and treatment‐dependent changes in apparent cortical mechanical response.

The small difference in modulus between the nucleus and cytosol suggests that changes in cortex stiffness cannot be attributed solely to intracellular structures such as the nucleus. Instead, it appears to be influenced by morphological changes on the cell surface, including the distribution of microvilli. In the cortical region, stiffness regulation can occur not only from increased surface roughness driven by microvilli but also from remodeling of actin microfilaments, which are critical for maintaining cell shape and facilitating intracellular transport. Previous studies have shown that spread area and traction force directly affect cortex thickness, with reduced adhesion leading to increased cortex thickness [75]. The combination‐treated group exhibited reduced cell adhesion strength following decreased focal adhesion, which may have promoted cortex thickening, thereby increasing surface roughness and cortical stiffness. In support of this, cytoskeletal network metrics in Figure 2 revealed the highest actin intensity in the cortex combination‐treated cells, closely correlating with the elevated Sneddon modulus observed. Overall, drug‐induced decreases in cell area and perinuclear actin cap tension appear to drive actin accumulation in the cortical region, leading to increased stiffness. The schematic diagram (Figure 5g) illustrates the mechanistic interplay between microvilli structure, actin cytoskeletal distribution, and cellular mechanical properties across treatment groups. This visualization demonstrates how surface structural changes coordinate with intracellular cytoskeletal dynamics to define the mechanical phenotype of cancer cells. Finally, the circular correlation heatmap analysis (Figure 5h) revealed that bearing area, cytosolic and nuclear modulus, and surface roughness were all negatively correlated with cell invasiveness. This finding supports the core hypothesis of this study: that increased structural complexity and stiffness at the cell surface diminish motility, underscoring the importance of mechanical properties as predictive indicators of cancer cell invasiveness.

### 2.6 Mechanomics‐Based Multivariate Analysis Reveals Key Biophysical Drivers of Therapeutic Response

**FIGURE 6:**
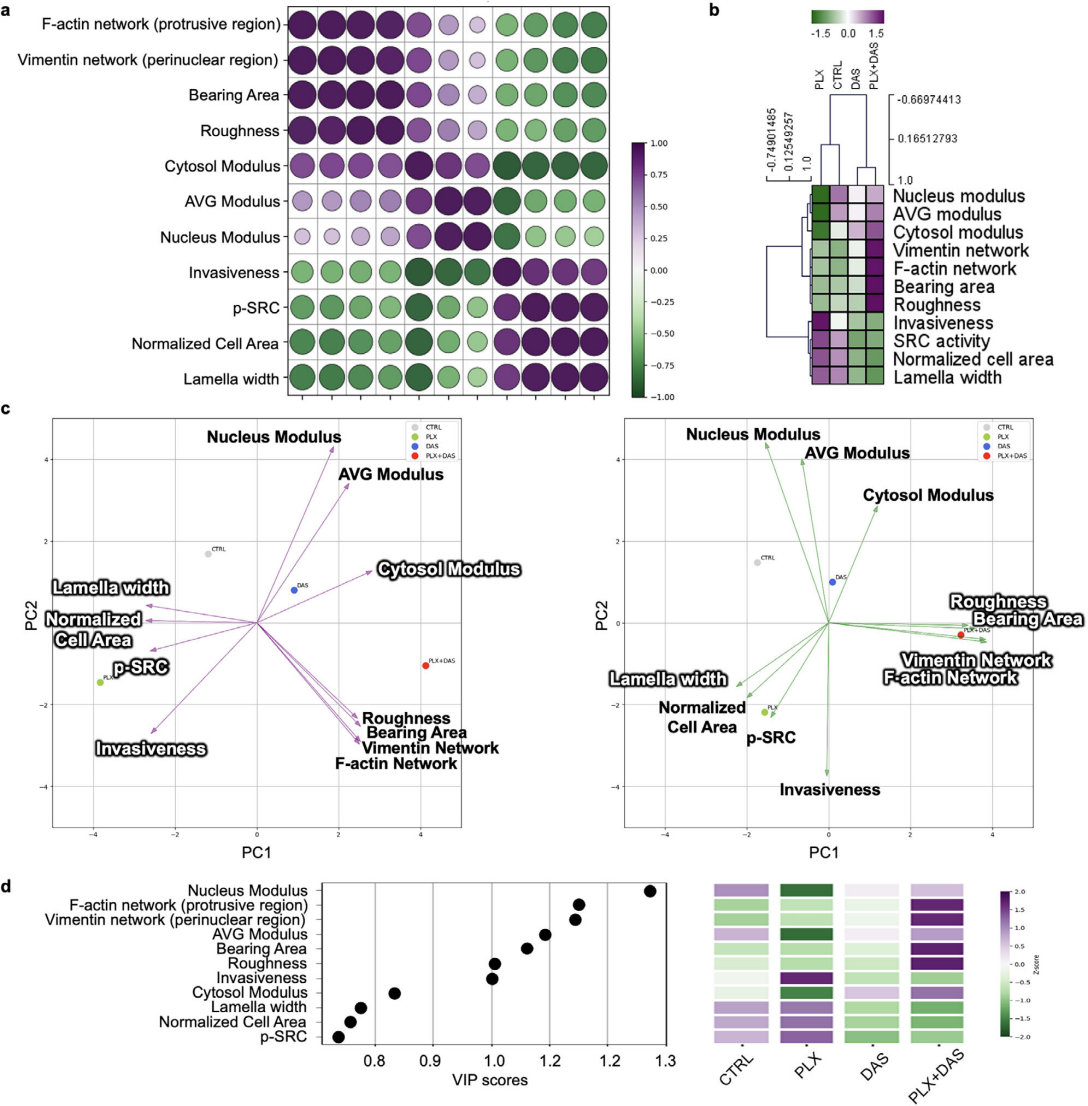
Mechanomics‐guided multidimensional analysis uncovers biophysical markers of therapeutic response. (a) Circular correlation matrix depicting strong positive associations among cytoskeletal and surface‐related parameters (F‐actin network density, vimentin architecture, bearing area, and surface roughness), and inverse correlations with stiffness‐associated features (nuclear modulus, cytosolic modulus, and average modulus). (b) Mechanomics‐driven hierarchical clustering reveals a treatment‐specific mechanophenotype under therapeutic coinhibition, featuring increased cortical stiffness and suppressed cytoskeletal dynamics. (c) PCA (left) and PLS‐DA (right) analyses based on mechanomics data identify principal biophysical markers defining treatment‐specific mechanophenotypes. (d) VIP scores rank the relevance of each mechanophenotypic trait in contributing to group discrimination, aligned with z‐scored heatmap visualization.

Altogether, we identified 11 key factors closely associated with metastatic potential by an integrated analysis of cellular structural and mechanical datasets (Figure S7a). To comprehensively interpret these biomechanical responses, we employed a hierarchical analytical framework encompassing correlation matrix analysis, multivariate dimensionality reduction, and quantitative variable importance projection (VIP) scoring. The circular correlation heatmap revealed that features such as the F‐actin network, vimentin network, bearing area, and surface roughness exhibited strong positive correlations and formed functional clusters along a common axis (Figure 6a). This clustering pattern underscores a mechanistic relationship between cytoskeletal remodeling and surface complexity in invasive phenotypes. In particular, SRC activity and invasiveness displayed a strong negative correlation with the cytoskeletal features, suggesting a potential inverse relationship between surface complexity‐induced stiffness and proinvasive signaling pathways.

Hierarchical clustering analysis demonstrated that cells subjected to PLX and DAS co‐treatment formed a distinct mechanophenotypic cluster, separate from those treated with PLX or DAS monotherapy (Figure 6b). This combination group showed coordinated modulation of stiffness‐related traits, including increased modulus values, concomitant with suppressed cytoskeletal protrusions and reduced surface complexity. These simultaneous alterations paralleled a pronounced reduction in cellular invasiveness, reinforcing the functional link between cytoskeletal architecture, mechanical remodeling, and metastatic potential.

To further dissect the contribution of individual features, we performed principal component analysis (PCA) and partial least squares discriminant analysis (PLS‐DA), which projected treatment‐dependent variance into low‐dimensional spaces (Figure S7b). The associated loading plots (Figure 6c) revealed that, in PCA, cytosolic modulus was the major contributor to separation along PC1, while nuclear modulus primarily defined variation along PC2 (Figure S7c). In contrast, PLS‐DA indicated that F‐actin network features strongly influenced cell separation along the PLS1 axis, while nucleus modulus was the primary determinant of cell separation along the PLS2 axis. Both biplots consistently showed that the PLX+DAS treatment group was distinctly segregated from other conditions. In particular, the biplot of PLS‐DA highlighted unique feature vectors in the combination treatment group, reflecting simultaneous inhibition of invasiveness along with increased cell stiffness and mechanical stability. These results suggest that the combination treatment induces a distinct mechanophenotype not observed in either monotherapy or control groups.

To quantify the relative contribution of each trait, we calculated VIP scores, overcoming the limitations of relying solely on feature loadings (Figure 6d). The VIP score quantifies the relative importance of each feature by integrating its classification contribution and its cross‐correlations with other variables. This analysis identified nucleus modulus, F‐actin network, vimentin network, average modulus, and bearing area as the top five VIP features. Especially, significant z‐score shifts in these features within the combination treatment group highlight two mechanistic regulatory axes. The first involves increased cortical stiffness driven by nuclear lamina reinforcement, and the second reflects suppression of protrusive cytoskeletal structures, both functionally associated with reduced invasiveness. Through this hierarchical integrative analysis, we demonstrate that cytoskeletal and stiffness‐related features form a highly organized regulatory network. The observed patterns of nanomechanical reorganization serve as critical functional indicators for predicting metastatic potential and drug responsiveness. In conclusion, the integrative nanomechanical profiling presented here identifies mechanical biomarkers linked to therapeutic efficacy and offers a powerful analytical platform to elucidate the interplay between cytoskeletal organization, mechanical properties, and cellular functions.

### 2.7 Mechanomics‐Based Integration of Cytoskeletal Organization and Nanomechanical State Defines Cancer Cell Invasiveness

**FIGURE 7:**
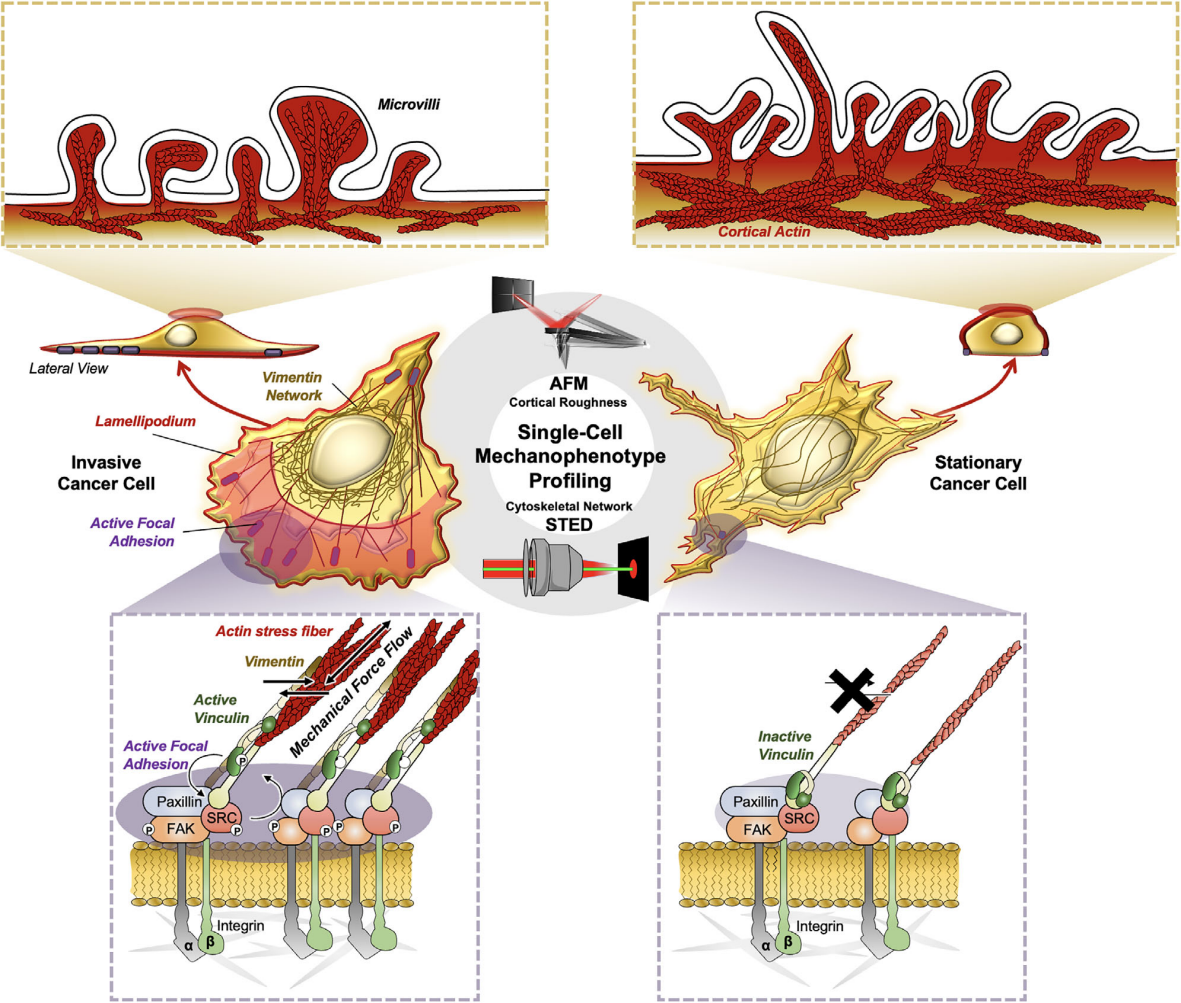
Multimodal single‐cell mechanomics integrating AFM‐based cortical measurements and cytoskeletal structure analyzed by STED demonstrates how therapeutic perturbations reconstruct cytoskeletal structure and mechanophenotypes. Invasive cancer cells exhibit distinct lamellipodia formation, activated cell adhesion signaling, and reduced microvilli‐related surface roughness, which correlate with decreased stiffness and increased motility. Conversely, treatment inhibits cancer cell invasion by increasing cortical stiffness, irregularizing microvilli structure, and disrupting the actin–vimentin network.

Figure 7 conceptually summarizes the interrelationships between cytoskeletal organization and mechanical properties identified in this study. In the left image, PLX‐treated cells (invasive cancer cells) show a flat morphology with extended lamellipodia and a directional organization of actin stress fibers and vimentin networks. Active SRC/FAK signaling promotes focal adhesion formation and vinculin activation, facilitating rapid transmission of mechanical forces toward the cell front. This suggests that the cells have transitioned into a more flexible and highly metastatic state. Nonlinear and less prominent microvilli, reduced surface roughness, and low cortical stiffness indicate an invasive phenotype with increased mechanical sensitivity and motility. In contrast, combination‐treated cells (stationary cancer cells) exhibit a shrunken and rounded morphology, with suppressed lamellipodia formation and impaired alignment and connectivity of actin and vimentin networks. Focal adhesion formation is inhibited, vinculin remains inactive, and SRC/FAK signaling is reduced. Consequently, force transmission to the cell front is diminished, maintaining the cells in a less motile and less invasive state. Short and irregular microvilli on the surface indicate enhanced surface roughness and stiffness, which likely contribute to cytoskeletal stabilization and suppressed invasiveness. Therefore, combination treatment with DAS effectively suppresses PLX‐induced mechanical sensitivity and motility. It is considered to reorganize the cytoskeletal structure and weaken connectivity, thereby increasing mechanical rigidity and ultimately reducing invasiveness. Thus, cytoskeletal remodeling and changes in mechanical properties constitute key mechanisms regulating cancer cell invasiveness.

## 3 Conclusion

To understand tumor progression and metastasis, an integrated perspective is required that incorporates not only molecular signaling but also the physical characteristics of cancer cells. Cytoskeletal remodeling in response to tumor‐inducing signals or treatment results in changes in cortical stiffness, surface roughness, and mechanical responsiveness, which are factors determining cancer cell invasiveness. However, previous studies have largely overlooked how drugs directly reprogram these mechanical properties. Here, we demonstrate that therapeutic perturbation induces a proinvasive mechanophenotype via activation of a resistance mechanism, while co‐treatment reverses this shift. By combining AFM‐based single‐cell nanomechanical profiling with STED imaging, immunoblotting, and morphodynamic analysis, we linked microvilli remodeling, cytoskeletal disorganization, and altered force transmission structures to invasive potential. Drug‐induced resistance response reduced cortical stiffness and promoted cell polarity and focal adhesion activation, indicative of enhanced motility. In contrast, appropriate combination therapy induced rounded morphology, suppressed lamellipodia and focal adhesion structures, and elevated cortical stiffness. Comparison of mechanical properties, including Sneddon modulus and surface roughness, at the single‐cell level under standardized fixed‐cell measurement conditions revealed that stiffness‐related indicators predicted treatment‐related mechanical phenotypic changes, which was consistent with imaging and molecular data.

In conclusion, our multimodal biophysical analysis reveals that cytoskeletal and mechanical reprogramming under targeted therapy critically shapes cancer cell invasiveness. AFM‐based single‐cell nanomechanical profiling provides a robust framework for analyzing how mechanophenotypes evolve under treatment and demonstrates its potential as a complementary strategy to conventional molecular assays in identifying predictive biomechanical markers of therapeutic response. Nevertheless, the AFM‐derived modulus values in this study should be interpreted as relative mechanophenotypic readouts obtained under standardized fixed‐cell measurement conditions, rather than absolute representations of cellular mechanics at physiological temperature. Future studies using a temperature‐controlled live‐cell AFM chamber will enable more accurate assessment of the physiological relevance of these treatment‐induced nanomechanical changes. Moreover, although identical substrate and coating conditions were used across all experimental groups to minimize ECM‐related variability, ECM composition and substrate stiffness may influence treatment‐related migratory and invasive phenotypes. Therefore, we intend to extend our systematic mechanophenotype profiling strategy to physiologically relevant ECM models, mechanically tunable substrates, various cancer models, and multicellular systems incorporating stromal and immune components, to further evaluate the generalizability of these mechanophenotypic transitions and their relevance to the tumor microenvironment.

## 4 Experimental Section

### 4.1 Cell Culture

Human thyroid cancer cell line (8505C) with BRAF^V600E^ mutation was obtained from DSMZ (German collection of microorganisms and cell cultures, Braunschweig, Germany) and cultured with RPMI1640 media (11874‐119, Gibco, Carlsbad, CA, USA) supplemented with 10% FBS (FBS; 10082–147, Gibco) and 1% antibiotic–antimycotic (AA; 15240‐062, Gibco) in a humidified incubator containing 5% CO_2_ at 37°C.

### 4.2 Primary Antibodies

Vimentin (5741), SRC (2108), p‐SRC (Tyr416; 2101), non p‐SRC (Tyr416; 2102), FAK (3285), p‐FAK (Tyr397; 3238), and p‐FAK (Tyr925; 3284) were purchased from Cell Signaling Technology (Beverly, MA, USA). Vinculin (ab18058) and Paxillin (ab32084) were acquired from Abcam (Cambridge, MA, USA). p‐Vinculin (Tyr1065; 44–1078G) was obtained from ThermoFisher Scientific (Waltham, MA, USA). β‐actin (sc‐47778) was acquired from Santa Cruz Biotechnology (Dallas, TX, USA), and F‐actin (Actin‐stain 555 phalloidin; PHDH1) was purchased from Cytoskeleton, Inc. (Denver, CO, USA).

### 4.3 Secondary Antibodies

Goat Anti‐Rabbit/Mouse IgG H&L (Alexa Fluor488) (ab150077/ab150113) and Donkey Anti‐Rabbit/Mouse IgG H&L (Alexa Fluor555, ab150074/ab150106) were purchased from Abcam. The HRP‐conjugated anti‐rabbit IgG or anti‐mouse IgG secondary antibodies used for immunoblotting analysis were from Cell Signaling Technology. Abberior STAR 488 and Abberior STAR 580 (Abberior GmbH, Göttingen, Germany) were used for STED imaging.

### 4.4 Inhibitors

PLX4032 (BRAF inhibitor, Vemurafenib; S1267) and dasatinib (SRC inhibitor; S1021) were obtained from Selleckchem (Houston, TX, USA).

### 4.5 Cell Viability Evaluation by Colony Formation Assay

To evaluate the inhibitory effects of compounds, cells (0.5 × 10^5^ or 1 × 10^5^ cells per well) were plated on a 24‐well plate. After 24 h, the cells were exposed to 1 or 5 µm PLX4032 and 5 µm dasatinib in combination for 6, 12, and 24 h. The cells were fixed with 4% paraformaldehyde (P2031, Biosesang, Seongnam, Korea) for 15 min and stained with 1% crystal violet solution‐0.25% methanol in distilled H_2_O (V5265, Sigma‐ Aldrich, St. Louis, MO, USA) and quantified using ImageJ software. Data were shown as mean ± standard deviation (*n* = 4).

### 4.6 Western Blot

Total protein was extracted using 1X RIPA buffer (10X; 9806, Cell Signaling) supplemented with 1 mm PMSF (200 mm; 8553, Cell Signaling) and 1X Protease/Phosphatase Inhibitor Cocktail (100X; 5872, Cell Signaling). Protein concentration was determined by the Pierce BCA Protein Assay Kit (23227, ThermoFisher Scientific) according to the manufacturer's instructions. Proteins were separated by 8% to 12% sodium dodecyl sulfate– polyacrylamide gel electrophoresis (SDS‐PAGE) gel (S2002, Biosesang) and transferred to an Immobilon‐P PVDF membrane (IPVH00010, Millipore, Bedford, MA, USA) using standard techniques. Each membrane was blocked with 5% Non‐Fat Powdered Milk (N1059, Biosesang) in 1X TBST (10X; T2006, Biosesang) and incubated with the primary and HRP‐linked secondary antibodies. The signals were detected with Immobilon Western Chemiluminescent HRP Substrate (WBKLS0500, Millipore), and Blots were exposed in ImageQuant LAS‐4000 mini (GE Healthcare Life Sciences, Pittsburgh, PA, USA).

### 4.7 In Situ Single‐Cell Nanoscoping

Cells (5 × 10^5^ cells per dish) were seeded into a WillCo‐dish Glass Bottom dish, Series GWST‐5040 (WillCo Wells B.V., Amsterdam, NL), in RPMI1640 supplemented with 10% FBS and 1% AA, and treated the following day with PLX4032 and dasatinib. The cells were grown for 2 days to a final confluence of 50–70%, then fixed with 4% paraformaldehyde for 15 min and gently washed with DPBS. Before nanoscopic measurements, DPBS was replaced with HEPES buffer (Gibco) to maintain pH stability under ambient CO_2_ conditions and minimize buffer‐related variability during AFM measurement. Atomic force microscopy was performed at room temperature in PeakForce QNM in Fluid mode using a BioScope Resolve AFM (Bruker Nano Surfaces, Santa Barbara, CA, USA) system mounted on an AXIO Observer A1 inverted optical microscope (Carl Zeiss, Germany). PeakForce QNM Live Cell Probe A, calibrated probe (PFQNM‐LC‐A‐CAL; Bruker AFM Probes, Camarillo, CA, USA) (Spring constant: 0.1 N m^−1^, Tip length 18 µm, Tip radius 70 nm, Tip half angle 18°) was used to image the cell surface. The Sneddon modulus for conical indenter was used for indentation analysis of cell surface:

$$F=\frac{2}{\pi }\frac{E}{\left(1-\upsilon^{2}\right)}\tan\left(\alpha \right)\delta^{2}$$

where *F* is the force acting on the cantilever tip (from force curve, nN); *δ* is the indentation depth (nm); υ is the Poisson's ratio (sample dependent, typically 0.2–0.5); *α* is the half‐angle of the indenter, and *E* is Young's modulus (fit parameter, kPa). The Height sensor, PeakForce Error, and Sneddon modulus signals were used to display the cell surface profiles using Nanoscope Analysis v1.60 (Bruker Nano Surfaces, Santa Barbara, CA, USA).

### 4.8 Transmission Electron Microscopy

Cells (1 × 10^6^ cells) grown in 10% RPMI1640 medium were fixed for 24 h in Karnovsky's fixative (2% Glutaraldehyde, 2% Paraformaldehyde, 0.5% CaCl_2,_ followed by 0.1 m phosphate buffer (pH 7.4)). Ultrathin sections were cut by LEICA EM UC‐7(Leica Microsystems, Austria) with a diamond knife (Diatome) and were double‐stained with 6% uranyl acetate (EMS, 22 400 for 20 min) and lead citrate (Fisher, for 10 min) for contrast staining. The sections were transferred on copper and nickel grids and observed by TEM (Transmission Electron Microscope: JEM‐1011, JEOL, Japan) with Camera‐Megaview III (soft imaging system, Germany) at an acceleration voltage of 80 kV.

### 4.9 Immunofluorescence Staining for Confocal and STED Microscopic Imaging

Cells (1 × 10^5^ cells per well) were seeded on round‐shaped glass coverslips in a 4‐well plate. After 24 h of incubation, the cells were treated with 5 µm PLX4032 and dasatinib inhibitors individually or in combination for 6 h. The cells were fixed for 30 min with 4% paraformaldehyde (Biosesang, Korea) and washed three times with 1X PBS (Welgene Inc., Korea). For permeabilization, 0.05% Triton X‐100 (T9285, Sigma Aldrich) was treated to cells for 15 min and blocked with 1% bovine serum albumin (BSA; BSAS‐NZ, Bovogen, VIC, Australia) and 0.001% sodium azide (26628‐22‐8, JUNSEI chemical, Tokyo, Japan). The cells were stained with primary antibodies (1:200) and secondary antibodies (1:500). Hoechst33342 (ThermoFisher Scientific) was added for nucleus staining at a final concentration of 5 mg mL^−1^. The confocal microscopic images were captured by LSM700 (Carl Zeiss, Jena, Germany), and the ZEN software was used for analysis. For super‐resolution STED imaging, cell images were acquired by a STEDYCON (Abberior Instruments GmbH, Göttingen, Germany) mounted at the camera port of an IX83 inverted microscope (Olympus, Tokyo, Japan) equipped with a 100x objective lens (UPLSAPO100XO/1.4, WD 0.13). The STEDYCON consisted of three excitation‐wavelength lasers at 450, 594, and 640 nm and one pulsed STED laser at 775 nm. The skeletal network analysis toolkit was constructed for analyzing the intracellular network of the vimentin and actin filament by Fiji software (http://fiji.sc/; GitHub, San Francisco, CA, USA).

### 4.10 Gene Set Enrichment Analysis

To visualize the protein interaction relationship network, protein–protein interaction (PPI) network and subnetworks were analyzed and constructed using the online STRING (http://string‐db.org/) database.

### 4.11 Statistical Analysis

All results are expressed as mean ± SD, and Student's *t*‐test was performed to determine statistically significant differences between groups, and *p*‐values (<0.01 or 0.05) were considered statistically significant.

## Author Contributions

M.K. and J.Y. designed and supervised the study. M.K., J.K., and N.Y. performed the experimental investigation. M.K., J.K., N.Y., and J.Y. conducted data analysis. M.K. and J.Y prepared the figures and wrote the manuscript. M.K. and J.Y. supervised the project. M.K., H.K.B., and J.Y. acquired funds. All authors contributed to the research, editing, and approval of the manuscript.

## Funding

This work was financially supported by the National R&D Program through the National Research Foundation of Korea (NRF) grant funded by the Ministry of Science and ICT (Grant Nos. NRF‐ 2020R1A2C1101616, NRF‐2021R1A2C1009894, NRF‐2020R1C1C1007776, RS‐2026‐25468605).

## Conflicts of Interest

The authors declare that they have no conflicts of interest.

## Supporting information




**Supporting File**: advs76613‐sup‐0001‐SuppMat.docx.

## Data Availability

The data that support the findings of this study are available in the supplementary material of this article.
