# Supplementary material for: Systemic Nanomechanical Single‐Cell Profiling Reveals Mechanophenotype Transitions Under Therapeutic Perturbation
Source: Adv Sci (Weinh). 2026 Jul 17:e76613. Online ahead of print. doi: 10.1002/advs.76613 (PMC13379253; doi:10.1002/advs.76613)
Supplement: Supplementary file 1 — Supporting File: advs76613‐sup‐0001‐SuppMat.docx. [file ADVS-9999-e76613-s001.docx]

Supporting Information

Systemic Nanomechanical Single-Cell Profiling Reveals Mechanophenotype Transitions under Therapeutic Perturbation

*Minhee Ku*, Jinwon Kwon, Nara Yoon, Hyung Kwon Byeon, Jaemoon Yang**

**Table S1** Functional enrichment analysis with BRAF, SRC, and cytoskeleton-related proteins by STRING. List of a cellular component comprising KEGG pathways and GO terms in functional clusters

| **KEGG Pathways** | | | |
| --- | --- | --- | --- |
| *pathway* | *description* | *count in gene set* | *false discovery rate* |
| hsa04510 | Focal adhesion | 10 of 197 | 1.07e-16 |
| hsa04810 | Regulation of actin cytoskeleton | 9 of 205 | 2.37e-14 |
| hsa04012 | ErbB signaling pathway | 7 of 83 | 1.05e-12 |
| hsa04015 | Rap1 signaling pathway | 8 of 203 | 2.24e-12 |
| hsa05219 | Bladder cacner | 6 of 41 | 2.96e-12 |
| **Cellualr Component (GO)** | | | |
| *GO-term* | *description* | *count in gene set* | *false discovery rate* |
| GO:0005925 | Focal adhesion | 5 of 134 | 8.97e-07 |
| GO:0005912 | Adherens junction | 6 of 252 | 8.97e-07 |
| GO:0031252 | Cell leading edge | 6 of 371 | 1.44e-06 |
| GO:0005886 | Plasma membrane | 12 of 5159 | 3.43e-06 |
| GO:0005856 | Cytoskeleton | 8 of 2068 | 0.00012 |


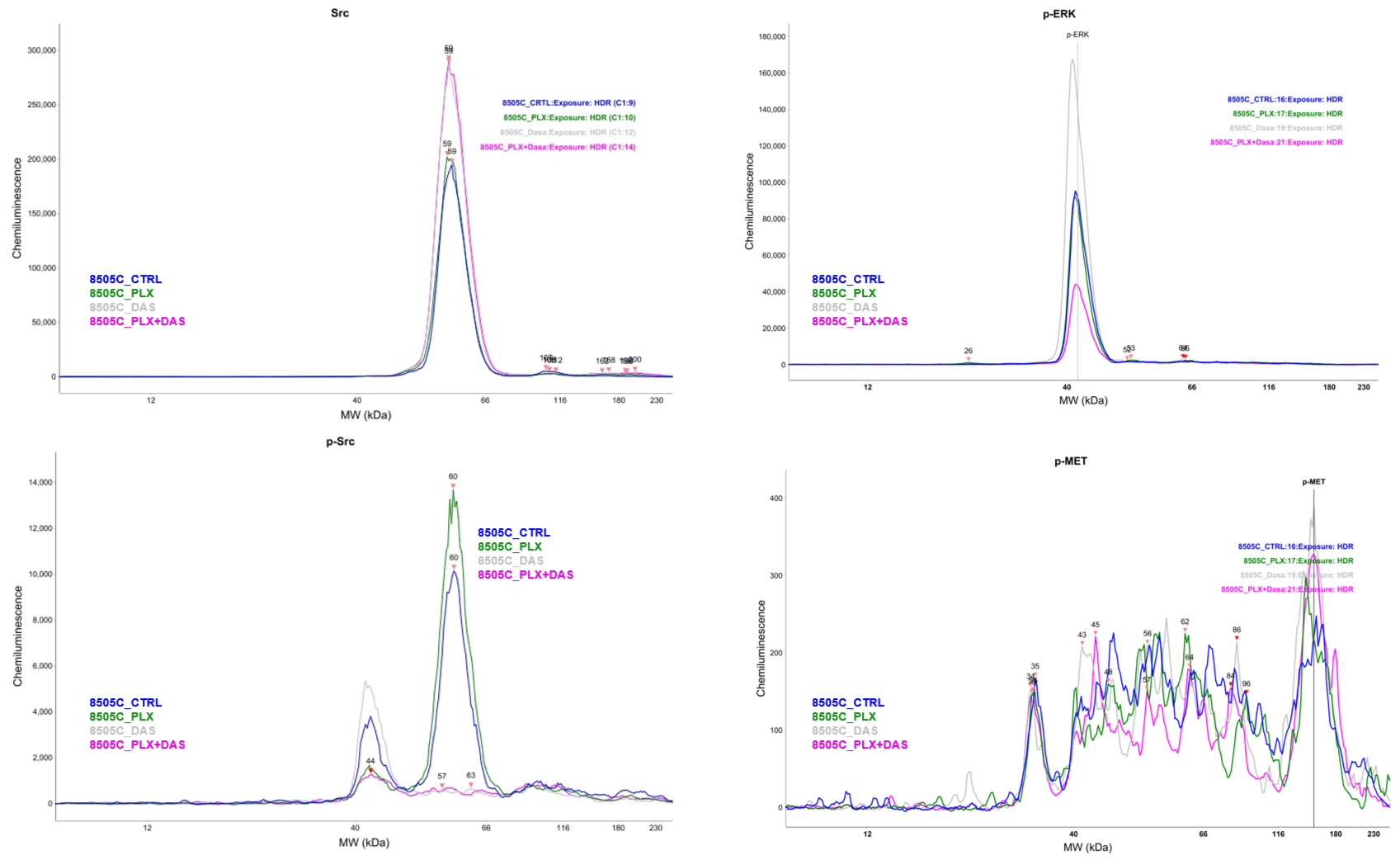


**Figure S1** The electropherogram representation by relative chemiluminescence measurements.


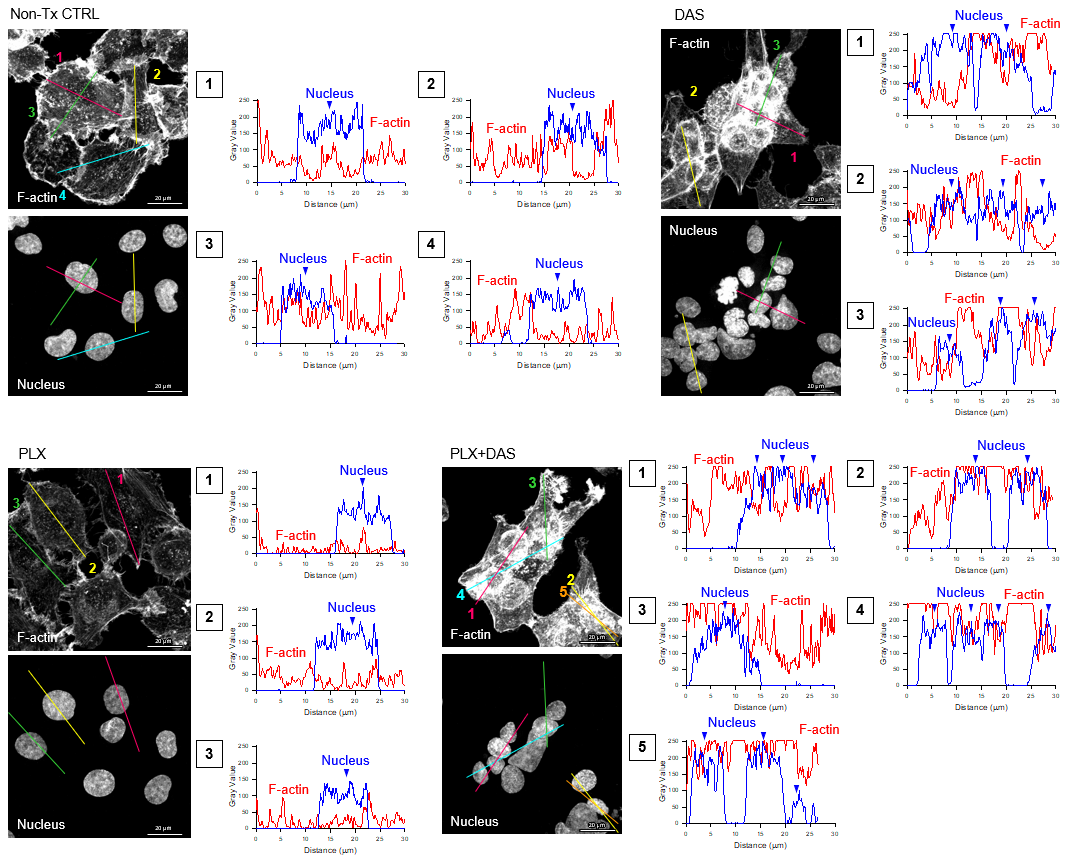


**Figure S2** Spatial intensity profiling of F-actin, nucleus, and vimentin across different treatment conditions. **a** Representative confocal images of F-actin and nuclei in cells treated with vehicle (CTRL), PLX4032 (PLX), dasatinib (DAS), or combination (PLX+DAS). Colored lines indicate scan paths used to quantify the spatial distribution of F-actin (red) and nuclei (blue). Corresponding line-scan plots show fluorescence intensity profiles of F-actin and nucleus along each line, revealing differences in cytoskeletal organization and nuclear positioning across conditions.


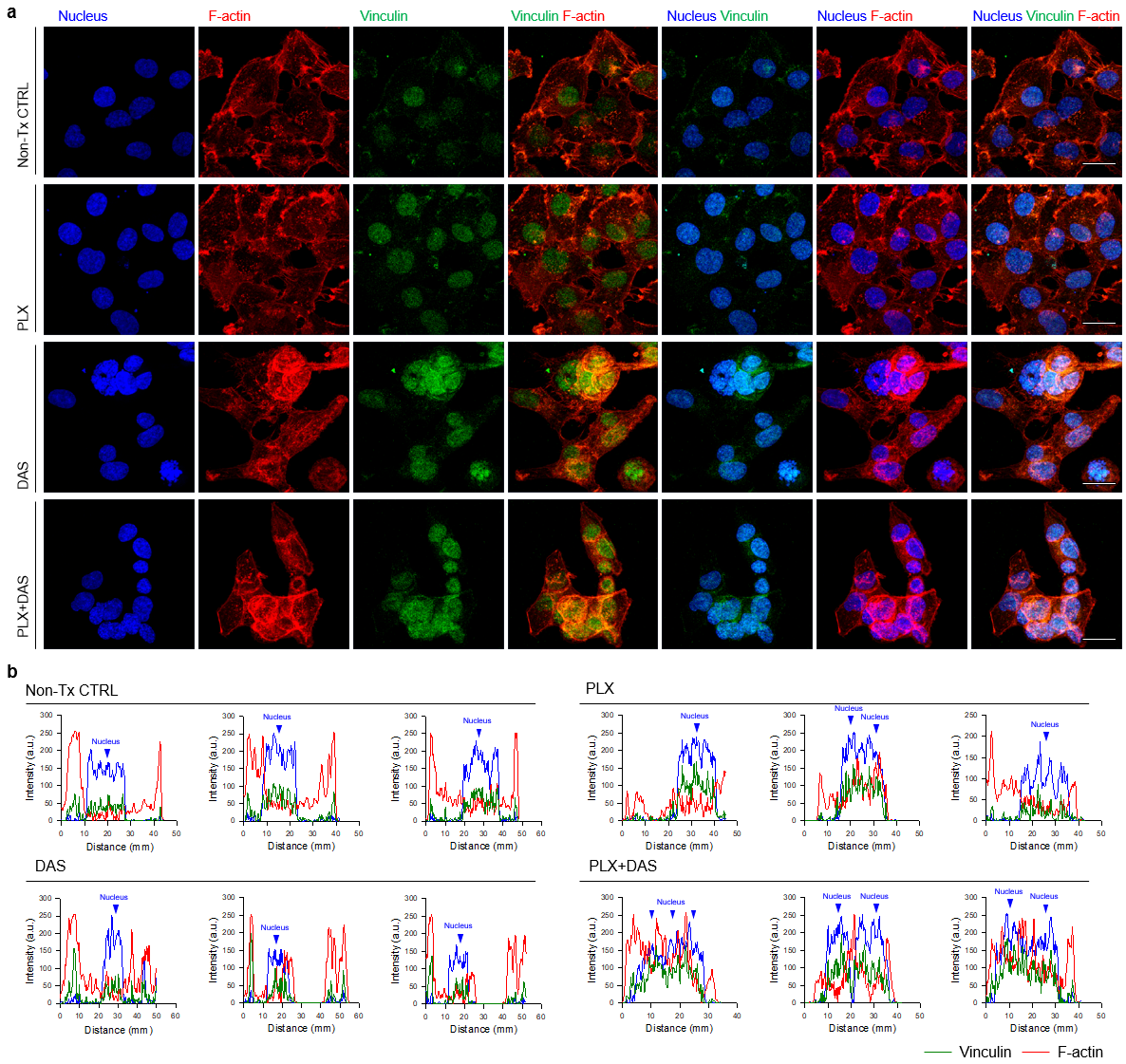


**Figure S3** Multi-channel confocal fluorescence images of vinculin and F-actin distribution under therapeutic perturbation conditions. **a** Representative confocal fluorescence images displaying the spatial distribution of nucleus (blue), vinculin (green), and F-actin (red) in 8505C cells under Non-Tx CTRL, PLX, DAS, and PLX+DAS treatment conditions. The full triple-channel overlays were used for subsequent orthogonal reconstruction and line-scan analyses presented in Figure 2b–c. Scale bars represent 20 μm. **b** Line profile analysis of F-actin (red), vinculin (green), and nucleus (blue) distributions. Each graph represents the fluorescence intensity (a.u.) plotted against distance (μm) for individual cells.


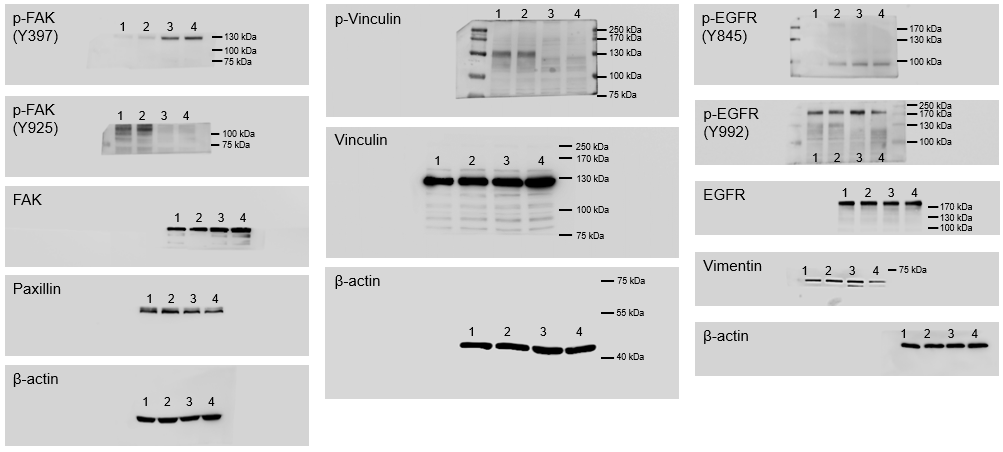
**Figure S4** Uncropped Western blot images corresponding to Figure 2d and Figure 3i. Full scan images of Western blots used to assess phosphorylation and expression levels of focal adhesion and EGFR signaling proteins, and vimentin under different treatment conditions: lane 1, vehicle (CTRL); lane 2, PLX4032 (PLX); lane 3, dasatinib (DAS); lane 4, combination (PLX+DAS). The blots corresponding to Figures 2d and 3i were obtained from the same membrane with identical sample lanes after sequential stripping and re-probing; therefore, a β-actin blot was used as the common loading control.


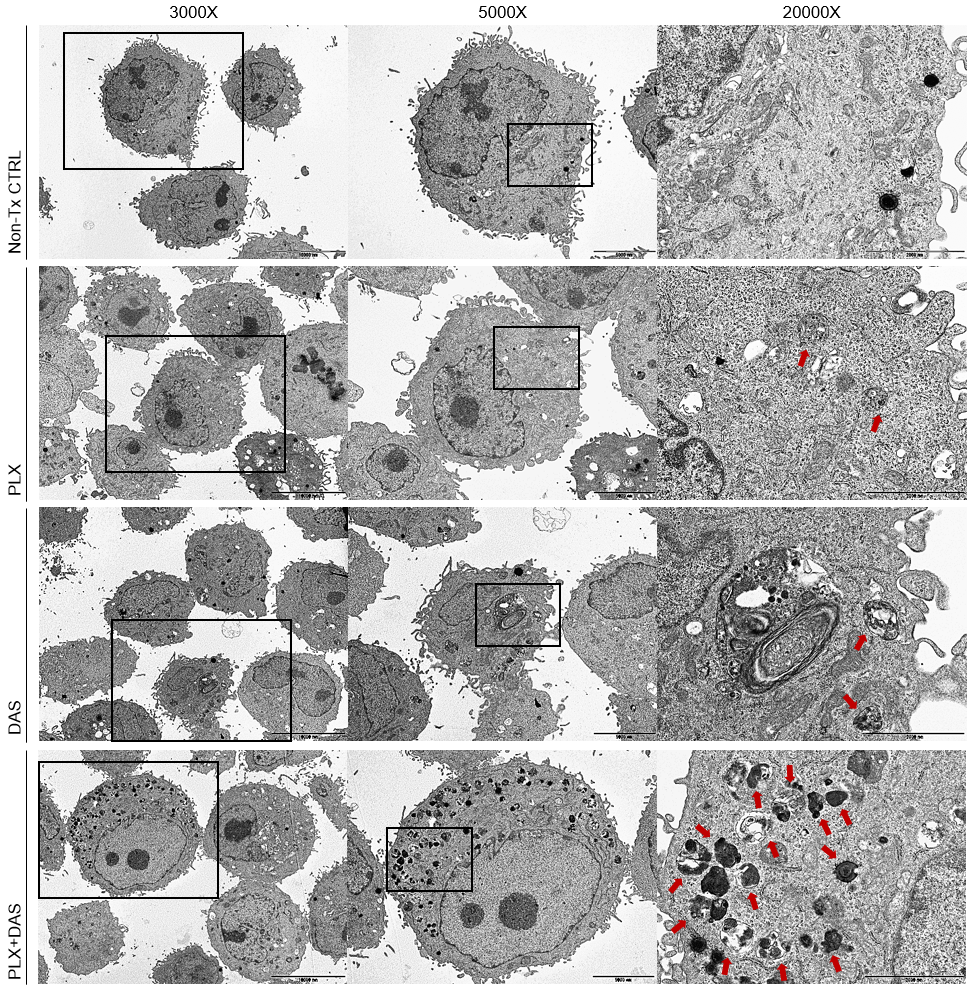


**Figure S5** Transmission electron microscopy (TEM) analysis of intracellular ultrastructural changes under different treatment conditions. Representative TEM images of cells treated with vehicle (CTRL), PLX4032 (PLX), dasatinib (DAS), or combination (PLX+DAS). Left and middle panels show low- and mid-magnification views highlighting overall cellular morphology. Right panels present high-magnification images of boxed regions, illustrating treatment-induced ultrastructural alterations. Red arrows indicate features associated with cell death, including increased autophagic vacuoles and electron-dense vesicles.


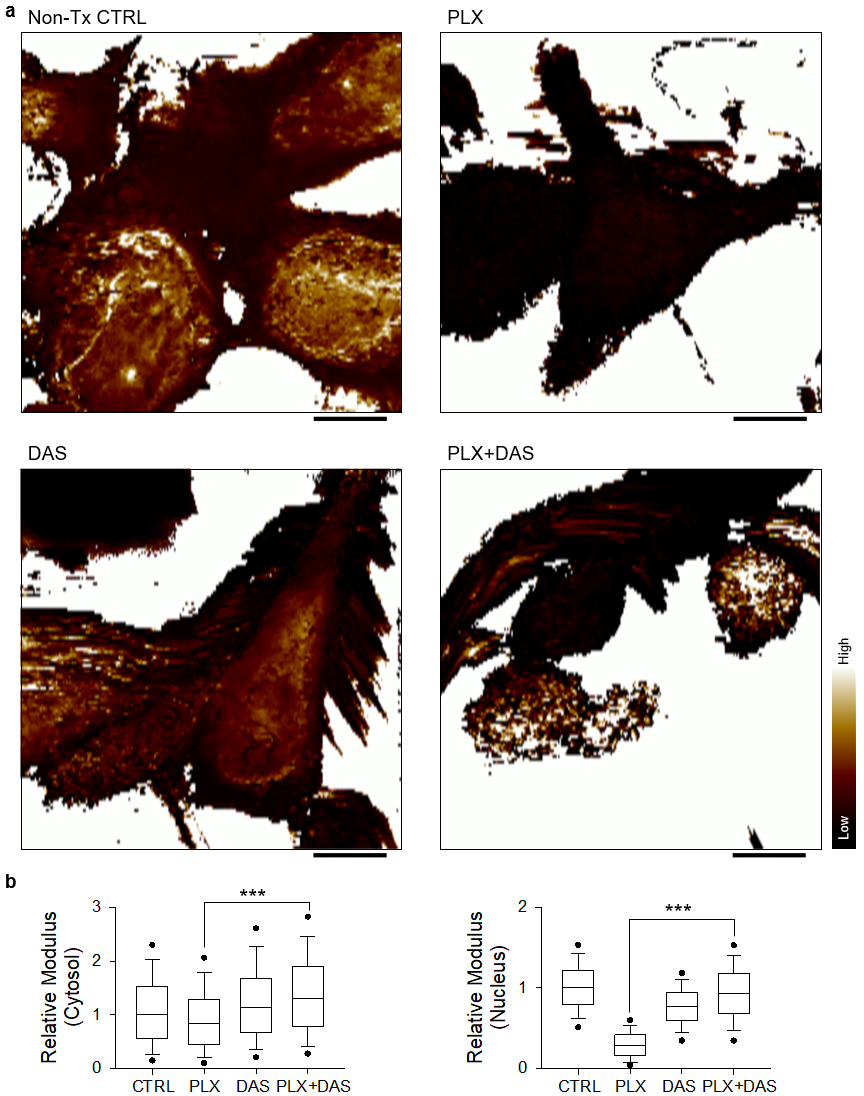


**Figure S6** Representative AFM results showing apparent Sneddon modulus contrast in fixed cells. **a** Representative Sneddon elasticity map acquired using PeakForce QNM of cells treated with vehicle (CTRL), PLX4032 (PLX), dasatinib (DAS), or combination (PLX+DAS) treatment. The maps were obtained from chemically fixed cells and present the Sneddon elasticity distribution under the same AFM measurement conditions. Scale bars, 10 µm. **b** Relative cytosol and nuclear apparent Sneddon modulus values were quantified from single-cell AFM measurements in each region and are shown as box-and-whisker plots. Statistical significance was evaluated using the Kruskal–Wallis test followed by Dunn’s multiple comparison test with Bonferroni correction. ***p < 0.001.


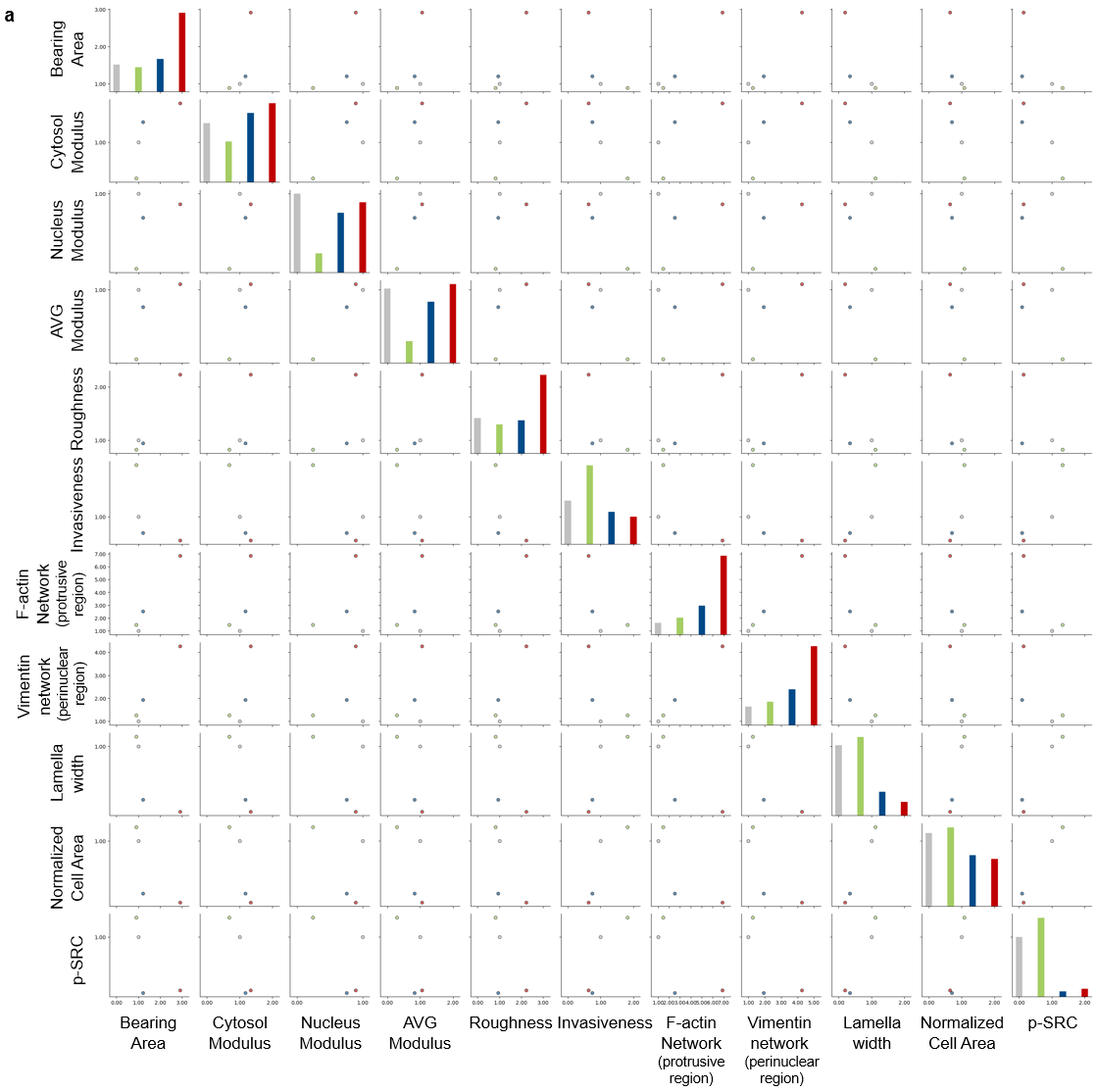


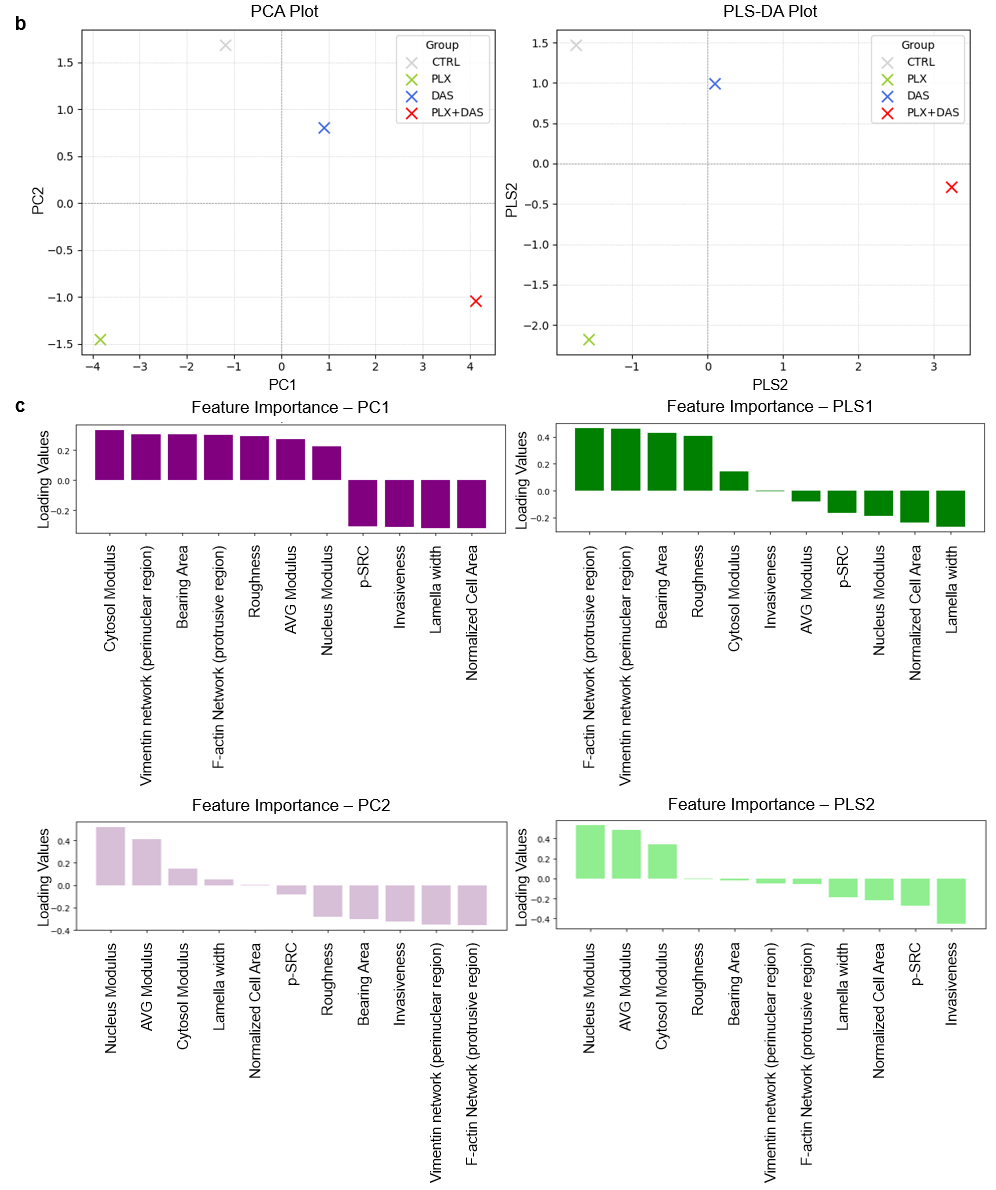


**Figure S7** Multivariate analysis of biophysical and structural features in treated cancer cells. **a** Pairwise correlation matrix of 11 normalized quantitative features, including modulus measurements (cytoplasmic, nuclear, average), cytoskeletal network intensities (F-actin, vimentin), cell shape descriptors (roughness, bearing area, cell area), and signaling activity (p-SRC). Diagonal histograms represent group-wise distributions, while scatter plots below the diagonal illustrate relationships between variables. **b** Principal Component Analysis (PCA, left) and Partial Least Squares Discriminant Analysis (PLS-DA, right) of the same 11 features. Each point represents a treatment group centroid (CTRL, PLX, DAS, PLX+DAS). **c** Feature importance plots for PC1/PC2 (left) and PLS1/PLS2 (right) dimensions.
